# Perioperative Magnesium for Postoperative Analgesia: An Umbrella Review of Systematic Reviews and Updated Meta-Analysis of Randomized Controlled Trials

**DOI:** 10.3390/jpm11121273

**Published:** 2021-12-02

**Authors:** Geun Joo Choi, Young Il Kim, Young Hyun Koo, Hyoung-Chul Oh, Hyun Kang

**Affiliations:** 1 Department of Anesthesiology and Pain Medicine, Chung-Ang University College of Medicine, Seoul 06911, Korea; pistis23@naver.com (G.J.C.); turtlebay7@naver.com (Y.I.K.); yhkoo28@caumc.or.kr (Y.H.K.); 2 Division of Gastroenterology, Department of Internal Medicine, Chung-Ang University College of Medicine, Seoul 06911, Korea; ohcgi@cau.ac.kr

**Keywords:** analgesia, magnesium, meta-analysis, pain, postoperative, systematic review, umbrella review

## Abstract

The purpose of this study was to summarize and evaluate evidence on the effectiveness of perioperative magnesium as an adjuvant for postoperative analgesia. We conducted an umbrella review of the evidence across systematic reviews and meta-analyses of randomized controlled trials (RCTs) on the effect of perioperative magnesium on pain after surgical procedures. Two independent investigators retrieved pain-related outcomes and assessed the methodological quality of the evidence of included studies using the A MeaSurement Tool to Assess systematic Reviews (AMSTAR) tool, and the Grading of Recommendations, Assessment, Development, and Evaluation (GRADE) system. In addition, an updated meta-analysis of postoperative pain-related outcomes with a trial sequential analysis (TSA) was conducted. Of the 773 articles initially identified, 17 systematic reviews and meta-analyses of 258 RCTs were included in the current umbrella review. Based on the AMSTAR tool, the overall confidence of the included systematic reviews was deemed critically low to low. Pain score, analgesic consumption, time to first analgesic request, and incidence of analgesic request were examined as pain-related outcomes. According to the GRADE system, the overall quality of evidence ranged from very low to moderate. While the updated meta-analysis showed the beneficial effect of perioperative magnesium on postoperative analgesia, and TSA appeared to suggest sufficient existing evidence, the heterogeneity was substantial for every outcome. Although the majority of included systematic reviews and updated meta-analysis showed a significant improvement in outcomes related to pain after surgery when magnesium was administered during the perioperative period, the evidence reveals a limited confidence in the beneficial effect of perioperative magnesium on postoperative pain.

## 1. Introduction

Postoperative pain can be linked to various pathophysiological pathways, including neuropathic and inflammatory pathways [1]. These various factors can be considered when developing a pain-management strategy following surgery. In this regard, there is a growing interest in the use of multimodal analgesia as an important component of ideal methods for postoperative pain management [2].

Magnesium has been shown to have analgesic benefits when used as an adjuvant in surgical patients and can therefore contribute to being part of a balanced analgesia strategy [3,4]. The regulation of calcium influx into the cell and antagonism of *N*-methyl-d-aspartate (NMDA) receptors in the central nervous system are assumed to be responsible for magnesium’s analgesic activity; however, the exact mechanism remains uncertain [5,6]. Numerous investigations have yielded contradictory findings since the first study on magnesium sulfate as an analgesic adjuvant in 1996 [4].

A number of systematic reviews and meta-analyses have been published that investigated the effect of magnesium administration during the perioperative period on postoperative pain. There has been no study to our knowledge that summarizes the evidence from these systematic reviews and meta-analyses. As a result, we re-evaluated the evidence in a systematic and comprehensive manner to offer an overview of the effect of perioperative magnesium administration on postoperative pain. We performed a qualitative umbrella review to analyze the findings of systematic reviews and meta-analyses that evaluated the efficacy of perioperative magnesium on postoperative pain as well as evidence of potential limits and consistency of results. We additionally conducted a quantitative meta-analysis including the latest data to update the existing evidence, which was also evaluated by trial sequential analysis.

## 2. Materials and Methods

An umbrella review summarizes previously published systematic reviews and meta-analyses to determine whether authors addressing comparable review issues have independently reported similar findings and reached comparable conclusions [7]. We conducted a literature review focusing on the effectiveness of perioperative magnesium as an adjuvant for postoperative pain. We also performed an updated meta-analysis including data from the last study. This study was prospectively registered with the PROSPERO database of systematic reviews (CRD42021265991) and conducted according to the Preferred Reporting Items for Systematic Reviews and Meta-Analyses (PRISMA) guidelines [8].

### 2.1. Search Strategy

We systematically searched MEDLINE, EMBASE, Cochrane Database of Systematic Reviews, and Scopus from inception to the end of March 2021 and updated on 1 July. A list of references from eligible systematic reviews and related reviews were scanned for additional citations. There were no language restrictions.

### 2.2. Eligibility Criteria

We included systematic reviews and meta-analyses reporting the effects of perioperative administration of magnesium as an adjuvant for postoperative pain. Two independent investigators (G.J.C. and Y.I.K.) assessed the following eligibility criteria: (1) Participants: adults and pediatric patients underwent surgery under general or regional anesthesia; (2) Intervention: magnesium administration as an adjuvant for postoperative pain management; (3) Comparison: placebo, no treatment, or no magnesium administration as control group; (4) Outcomes (summary measures for pain-related outcomes during postoperative period): pain score, analgesic consumption, time to analgesic request, and incidence of rescue analgesic; (5) Study design: systematic reviews and/or meta-analyses of randomized controlled trials (RCTs). In case of disagreement, all issues were discussed with a third investigator (H.K.). Narrative and other types of non-systematic reviews (e.g., critical reviews, overviews, state-of-the-art reviews), clinical practice guidelines, evidence summaries, critically appraised topics, clinical paths, consumer information sheets, best practice information sheets, technical reports, and other evidence-based pieces were excluded.

### 2.3. Data Extraction

Two investigators (G.J.C. and Y.I.K.) extracted the following from each eligible study: (1) first author, (2) year of publication, (3) name of journal, (4) number of included RCTs, (5) number of participants in each trial arm, (6) information on intervention and comparison, (7) surgical procedure, (8) information on outcomes reported as primary or secondary, and (9) type of effect size used in the meta-analysis (effect size with 95% confidence intervals (95% Cis)). We finally extracted the information required by the Assessment of Multiple Systematic Reviews (AMSTAR) tool [9].

### 2.4. Assessment of the Confidence and Quality of Evidence

The overall confidence of the included systematic reviews was determined using AMSTAR (A MeaSurement Tool to Assess systematic Reviews) version 2.0 [10]. This revised tool simplifies response categories and contains 16 items in all, which provide a more comprehensive appraisal compared with the original AMSTAR [9]. The quality of evidence for each pooled outcome from the included systematic reviews was assessed using GRADE (Grading of Recommendations Assessment, Development, and Evaluation) [11]. In this approach, the quality of evidence was categorized as high, moderate, low, or very low based on limitations in risk of bias, precision, consistency, directness, and publication bias.

AMSTAR evaluation and GRADE classification were independently performed by two investigators (G.J.C. and H.C.O.). Any discrepancy was resolved via discussion, and all discrepancies that could not be resolved through discussion were arbitrated by a third investigator (H.K.).

### 2.5. Updated Meta-Analysis

A study search was performed based on RCTs included in the systematic reviews of umbrella reviews and newly published RCTs since January 2020. RCTs were included in this updated meta-analysis if they compared perioperative magnesium administration with the control group in patients undergoing surgical procedures under anesthesia. There were no language restrictions, and postoperative pain-related outcomes were extracted. Two investigators (G.J.C. and Y.H.K.) independently performed the study search, study selection, and data extraction. In case of disagreement, all issues were discussed with a third investigator (H.K.).

### 2.6. Statistical Analysis

The pooled risk ratio (RR) and 95% confidence intervals (CIs) for incidence of rescue analgesic and standardized mean difference (SMD) and 95% CIs for pain score, analgesic consumption, and time to analgesic request were calculated, respectively. In terms of pain scores, visual analogue scale and numerical rating scale in adult patients and Children’s Hospital of Eastern Ontario Pain Scale in pediatric patients were utilized, respectively, which were standardized in outcome synthesis of updated meta-analysis. We used the chi-square test for homogeneity and the I^2^ test for heterogeneity. We regarded a level of 10% significance (*p* < 0.100) in the χ^2^ statistic or an I^2^ greater than 50% as considerable heterogeneity. For both categorical and continuous data, we used the DerSimnian–Laird random-effect model. Otherwise, we applied the fixed-effect model. As pain score data were measured at multiple time points, we adopted two strategies to select and analyze the data. First, we selected the data of the nearest time point from the specified time point: 0 h postoperatively for the PACU phase, 4 h postoperatively for the early phase, and 24 h postoperatively for the late phase. Second, as data measured at multiple time points were dependent on each other, and multiple comparisons at each time point would increase the possibility of type I error, we combined outcomes from multiple time points within specified periods (namely 0–1 h for PACU and 0–4 h for early phase) and performed the analysis using the pooled combined outcomes. Publication bias was evaluated using funnel plots and Egger’s linear regression test. If the funnel plot was asymmetrical, or the *p*-value was <0.100 by Egger’s test, we considered the presence of a publication bias and performed trim-and-fill analysis. 

As traditional meta-analysis runs the risk of random errors due to sparse data, we additionally performed a trial sequential analysis (TSA). TSA is a methodology that includes a required information size (RIS) calculation for a meta-analysis with a threshold for statistical significance, which controls the risk of potential false-positive and false-negative findings of meta-analyses and provides information on whether the results of our study were conclusive. We used a fixed or random effects model to construct a cumulative *Z*-curve. TSA was performed to maintain an overall 5% risk of type I error. Meta-analysis was conducted using Comprehensive Meta-Analysis version 2.0 (Englewood, NJ, USA, 2008) and TSA 0.9.5.10 β software (Copenhagen Trial Unit, Copenhagen, Denmark).

## 3. Results

### 3.1. Description of Included Systematic Reviews

**Figure 1:**
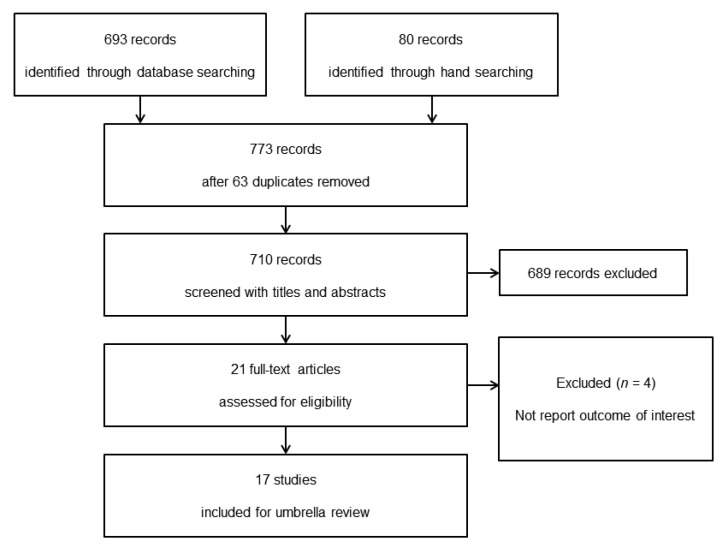
PRISMA flow chart of umbrella review.

Of the 773 articles initially identified, 21 full-text articles were assessed for eligibility. Four studies were excluded because there was no report regarding pain-related outcome during postoperative period [12,13,14,15]. Seventeen systematic reviews, including 258 individual RCT estimates, were finally selected for this umbrella review. The study selection process and reasons for exclusion are showed in Figure 1. Participants were pregnant women in two studies [16,17], pediatric patients in three [18,19,20], and adult patients in the others. Magnesium as an adjuvant was administered pre- and/or intraoperatively in the intervention group, while placebo or nothing in comparison with magnesium was administered in the control group. Sixteen included studies were systematic reviews and meta-analyses of RCTs, while one study was a systematic review of RCTs [21]. Routes of magnesium administration were intravenous in seven studies [21,22,23,24,25,26,27], intrathecal and/or epidural in five [16,17,28,29,30], and intra-articular in two [31,32]. There were several routes, including intravenous, intrathecal, epidural, and local approaches, in three studies [17,18,20]. The types of surgery were cesarean section in two studies [16,17], tonsillectomy in two [18,20], arthroscopic surgery in two [31,32], and laparoscopic cholecystectomy in one [23]. The remaining patients underwent multiple surgical procedures. The study characteristics of the adult and pediatric patients are summarized in Table 1.

### 3.2. Summary of the Evidences

Pain-related outcomes were pain score, analgesic consumption, time to first analgesic request, and number of patients requiring rescue analgesics after surgery. Ten studies in adult patients [16,17,22,23,24,25,26,27,31,32] and two studies in pediatric patients [18,20] conducted a meta-analysis regarding pain score after surgery. The majority of studies described using magnesium as part of a multimodal analgesic regimen to reduce postoperative pain intensity within 24 h of surgery. The pain score in adult patients showed a significant decrease in the magnesium group both at rest and during movement in a large body of meta-analyses, but heterogeneity was substantial. In contrast, the pain score in pediatric patients did not show any significant difference between the two groups. Summarized evidence on pain scores in adult and pediatric patients is shown in Table 2.

Ten studies conducted meta-analyses to evaluate the effect of magnesium on analgesic consumption [16,17,22,23,24,25,26,27,28,31]. All included studies showed a significant reduction in analgesic consumption after surgery when magnesium was administered, whereas the majority of included studies showed substantial heterogeneity. Summarized evidence on analgesic consumption is shown in Table 3.

Nine studies performed meta-analyses to assess the time interval to the first analgesic request following surgery [17,22,24,27,28,29,30,31,32]. Time to first analgesic request was significantly shorter in the majority of studies although heterogeneity was substantial; the details are presented in Table 4. 

In pediatric patients, the pain score did not show any significant difference between the two groups. Two studies performed meta-analyses on the incidence of rescue analgesics [19,20], which was significantly lower in patients who received magnesium. Summarized evidence on pain-related outcomes in pediatric patients is shown in Table 5.

### 3.3. Confidence and Quality of Evidence

Based on the AMSTAR 2.0 tool, the confidence of the included systematic reviews was deemed low to critically low. The quality of evidence on pain-related outcomes ranged from very low to moderate according to the GRADE system, with a large body of low-quality evidence. We assessed the quality of evidence on pain-related outcomes provided by each systematic review if there was no GRADE evaluation. They are summarized in Table 2, Table 3, Table 4, Table 5 for each outcome and patient category.

### 3.4. Results of Updated Meta-Analysis

Data of adult patients from 109 RCTs (of which two RCTs were newly included in this updated meta-analysis [33,34], and data of pediatric patients from 13 RCTs were extracted (supplementary Figure S1). In adult patients, pain scores at the PACU, early phase, and late phase showed more significant reductions in the magnesium group than in the control group (Table 6). The time to first analgesic was significantly longer in the magnesium group than in the control group (SMD = −1.867; 95% CI, −2.216 to −1.519; *p*_chi_^2^ < 0.001; I^2^ = 94.8%). Analgesic consumption was significantly reduced with the use of magnesium compared to the control group (SMD = 1.456; 95% CI, 1.163–1.749; *p*_chi_^2^ < 0.001; I^2^ = 94.7%).

**Figure 2:**
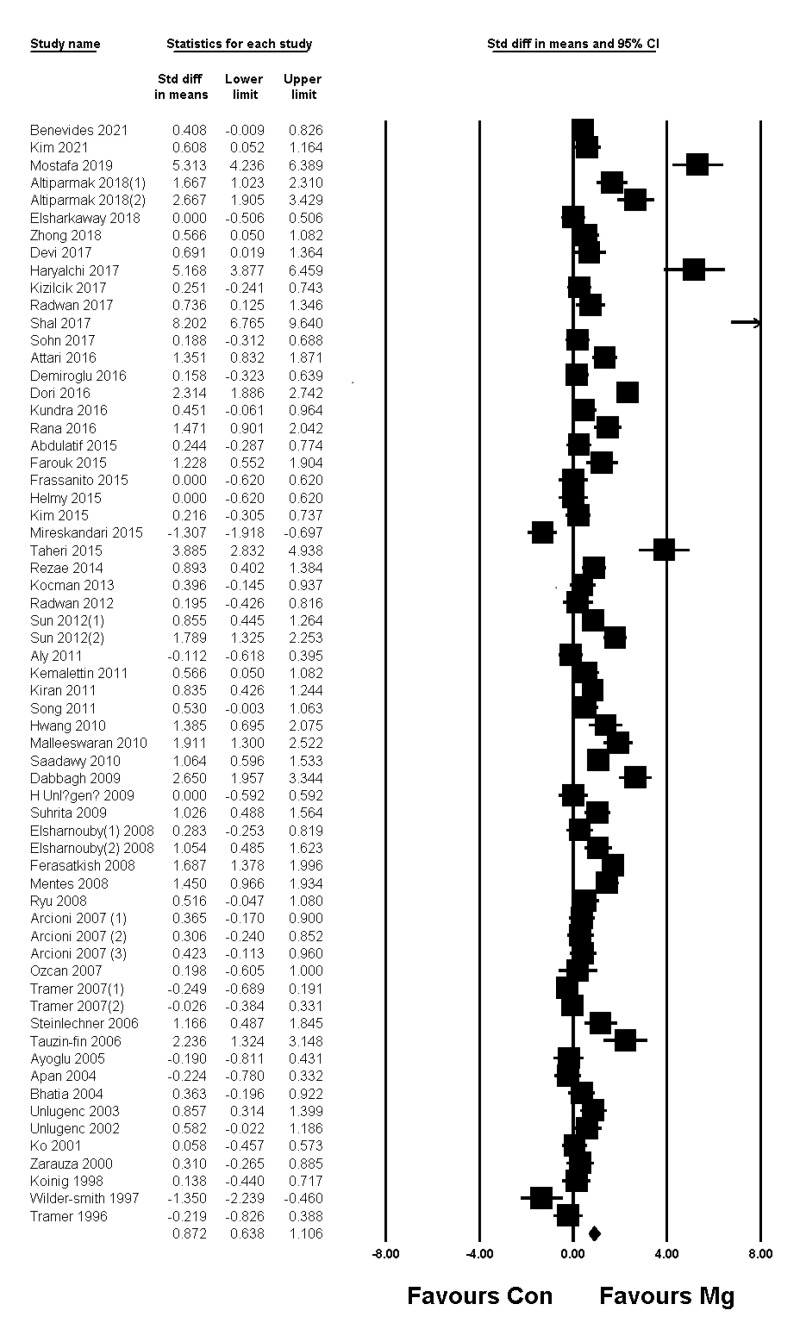
Forest plot showing pain score at postoperative 4 h in adult patients. The figure depicts individual trials as filled squares with relative sample size and the 95% confidence interval (CI) of the difference as a solid line. The diamond shape indicates the pooled estimate and uncertainty for the combined effect.

**Figure 3:**
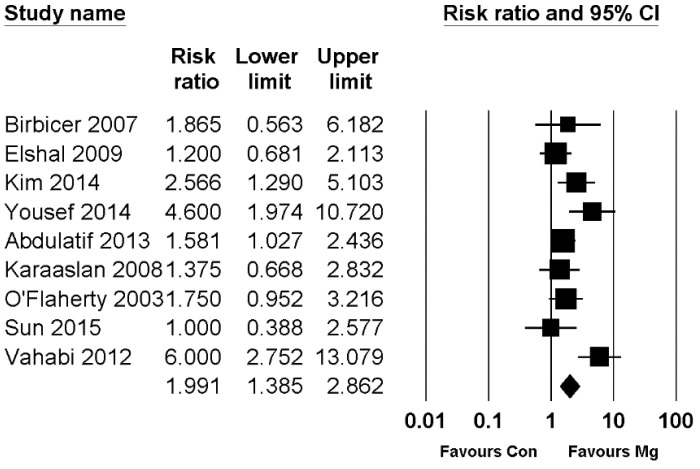
Forest plot showing the incidence of rescue analgesic in pediatric patients. The figure depicts individual trials as filled squares with relative sample size and the 95% confidence interval (CI) of the difference as a solid line. The diamond shape indicates the pooled estimate and uncertainty for the combined effect.

In pediatric patients, pain scores at the PACU and early phase showed significant reductions in the magnesium group compared to the control group, whereas the pain score at the late phase (at postoperative 24 h) did not show a significant difference between the two groups (Table 7). The time to first analgesic was significantly longer in the magnesium group than in the control group, and analgesic consumption and the incidence of rescue were significantly lower in the magnesium group than in the control group (Table 7). Forest plots of pain score at postoperative 4 h in adult patients and the incidence of rescue analgesic in pediatric patients are presented in Figure 2 and Figure 3, respectively. The other forest plots are shown in supplementary figures: adult resting pain score at 0 h, 0–1 h, 0–4 h, and 24 h in Figures S2–S5; adult dynamic pain score at 0 h, 0–1 h, 4 h, 0–4 h, and 24 h in Figures S6–S10; pediatric pain score at 0 h, 0–1 h, 4 h, 0–4 h, and 24 h in Figures S11–S15; and pediatric time to first analgesic and analgesic consumption in Figures S16 and S17.

**Figure 4:**
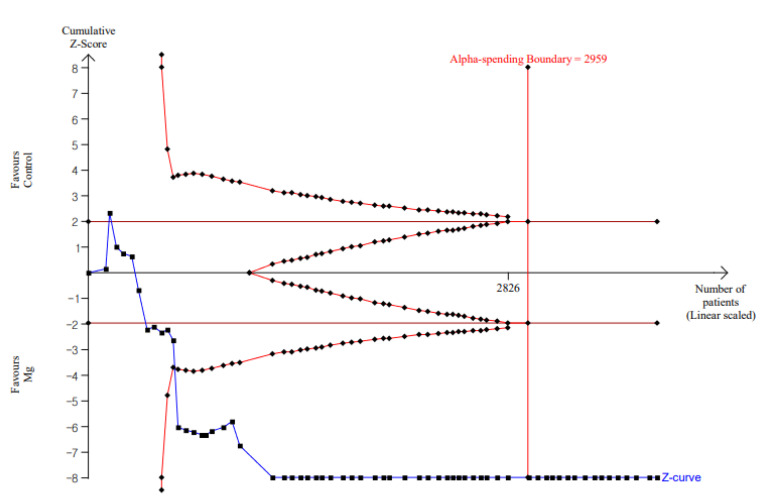
TSA for resting pain score at postoperative 4 h in adult patients. Complete blue line represents the cumulative Z curve, etched red line represents conventional test boundary, complete outer red line represents the trial sequential monitoring boundary, and complete inner red line represents futility boundary. The cumulative *Z* curve crossed the conventional test boundary and the trial sequential monitoring boundary.

TSA suggested that results of our updated meta-analysis were confirmative for each outcome (Table 6 and Table 7). Figure 4 shows the results of TSA of resting pain score at postoperative 4 h in adult patients. The other parameters with respect to the adult resting pain score at 0 h and 24 h are shown in Figures S18 and S19; adult dynamic pain score at 0 h, 4 h, and 24 h in Figures S20–S22; pediatric pain score at 0 h, 4 h, and 24 h in Figures S23–S25; and time to first analgesic, analgesic consumption, and incidence of rescue analgesic in Figures S26–S28. 

### 3.5. Publication Bias

A funnel plot was used for all comparisons. All displayed a symmetrical appearance for resting pain score at 0 h, 0–1 h, 4 h, 0–4 h, and 24 h (Figures S29–S33); adult dynamic pain score at 0 h, 0–1 h, 4 h, 0–4 h, and 24 h (Figures S34–S38); time to first analgesic (Figure S39) and analgesic consumption (Figure S40) in adults; and pediatric pain score at 0 h, 0–1 h, 4 h, 0–4 h, and 24 h (Figures S41–S45). The results of Egger’s test were as follows: adult resting pain score at 0 h (*p* = 0.170), 0–1 h (*p* = 0.266), 4 h (*p* = 0.212), 0–4 h (*p* = 0.103), and 24 h (*p* = 0.834); adult dynamic pain score at 0 h (*p* = 0.707), 0–1 h (*p* = 0.308), 4 h (*p* = 0.066), 0–4 h (*p* = 0.002), and 24 h (*p* = 0.064), time to first analgesic use (*p* < 0.001) in adults; analgesic consumption (*p* < 0.001) in adults; and pediatric pain score at 0 h (*p* = 0.114), 0–1 h (*p* = 0.235), 4 h (*p* = 0.734), 0–4 h (*p* = 0.175), and 24 h (*p* = 0.696). 

In terms of outcomes with *p*-value < 0.1 from Egger’s test, a trim-and-fill analysis was performed: adult dynamic pain score at 4 h (SMD = 0.942; 95% CI, 0.364 to 1.520) and at 0–4 h (SMD = 1.059; 95% CI, 0.561 to 1.556) and analgesic consumption in adults (SMD = −1.456; 95% CI, 1.163 to 1.749) showed significant changes after trim-and-fill analysis (SMD = 0.536; 95% CI, −0.120 to 1.191; SMD = 0.470; 95% CI, −0.082 to 1.022; SMD = 0.539; 95% CI, 0.226 to 0.852, respectively).

## 4. Discussion

Through this study, we provided a comprehensive overview of the reported effect of perioperative administration of magnesium on postoperative pain by incorporating evidence from meta-analyses and systematic reviews of RCTs and conducting an updated meta-analysis with TSA. The overall quality of evidence ranged from very low to moderate. We discovered that there was a large body of low-quality evidence that perioperative magnesium administration reduced the intensity of postoperative pain and diminished the need for postoperative opioid analgesia. In contrast, there is a small body of high-quality evidence supporting postoperative pain-related outcomes. Individual systematic reviews of this umbrella review included only RCTs as study designs. RCTs are initially given a high-quality rating, but after considering study limitations, indirectness, inconsistency, imprecision, and publication bias, they might be downgraded [35]. The possibility of bias and inconsistency can explain the current study’s predominantly low quality of evaluated outcomes. Various regimens, dosages, routes of magnesium administration, and different types of surgery can all have an impact on the quality of evidence based on the GRADE system.

An updated meta-analysis suggested that perioperative magnesium administration showed a significantly beneficial effect on postoperative analgesia, even with substantial heterogeneity. Indeed, as our understanding of the component of this receptor linked with pain pathophysiology has grown, the number of trials on systemic or local usage of magnesium has expanded in recent years. Magnesium modulates pain and inflammatory responses by blocking calcium channels and antagonizing the NMDA receptor, thereby reducing central sensitization to peripheral injury [5]. Moreover, there has been a growing interest in multimodal analgesia as an important component of appropriate perioperative pain-management strategy in patients undergoing surgery. Multimodal analgesia is defined as the use of various agents, primarily non-opioid analgesics, and non-pharmacologic therapies in combination to target a range of pain receptors in a synergistic manner [36]. Given its analgesic properties, magnesium can be a good option for multimodal analgesia. By assessing the overall quality and quantity of existing evidence in perioperative magnesium use for postoperative analgesia in this study, we may expect to narrow the gap between study evidence and its clinical applicability.

The findings of several recent trials regarding the use of perioperative magnesium are reassuring and have demonstrated the analgesic properties of magnesium in pediatric patients [18,20]. Unlike in adult trials, perioperative magnesium had no beneficial effect on pain intensity in pediatric patients in the current study. It is difficult to assess pain in children because they are unable or unwilling to express it. Even the Children’s Hospital of Eastern Ontario Pain Scale (CHEOPS), a pain rating system, is questionable because the ratings are often low and do not correspond well with self-reported measures [37]. In contrast, the considerable reduction in the incidence of rescue analgesia mediated by magnesium administration in this study may objectively suggest that perioperative magnesium administration can be useful in the pediatric group.

The overall confidence of the included systematic reviews was rated as critically low to low using AMSTAR 2.0 tool. The quality of evidence on pain-related outcomes reported in included studies ranged from very low to moderate based on GRADE system. Because of its multifaceted and subjective nature, pain is a complex clinical phenomena to quantify [38]. There is a general lack of uniformity in pain-related clinical trials, both regarding pain-related outcome assessment and description, making it difficult to synthesize data [39]. In this respect, the accuracy and practicality of objective pain measurements are advantageous. Analgesic consumption and incidence or time to first analgesia can be useful parameters as objective pain measurement tools in this regard, even if these outcomes exhibit significant heterogeneity. The analgesic effect of perioperative magnesium administration was examined in our study using subjective and objective measures, which could support its use for analgesic benefit. However, we should regard the low quality of evidence based on our assessment in this study, which means that more research will almost certainly have a considerable impact on confidence in the estimate of the effect and will certainly change the estimate. Given the inherent subjectivity of the GRADE approach, we performed TSA in addition to an updated meta-analysis. TSA can be thought of as a purely objective and quantitative approach [40], with the results indicating that current outcomes from existing research are sufficient and that therefore additional research may not be required. However, because sequential approaches have methodological limitations when heterogeneity is present, this result cannot be considered a powered conclusion [41]. When building on existing databases, TSA should be treated with caution because omissions and errors in interpretation and implementation may be common [42,43]. Hence, further research on the ideal amount of information and different types of interventions under diverse circumstances is needed to draw appropriate conclusions.

Given the wide variability in the methodology used in the included trials, this umbrella review has some limitations. Various surgical methods and anesthetic strategies were addressed in all the included studies. Although this is a standard method for quantitative systematic reviews of perioperative pain management, it is possible that this contributed to some of the observed heterogeneity. The routes of magnesium administration varied according to the anesthetic approach, contributing to high heterogeneity. This allowed for a more precise assessment of the magnesium analgesic effect, but it also limits the applicability of our findings to anesthetic practice when multimodal drugs are frequently used. Furthermore, in this study, we did not assess the safety of perioperative magnesium use, such as shivering, nausea, or vomiting following surgery. This will provide more information on the perioperative use of magnesium. Despite these limitations, our study has shown strength by including RCTs to present the first umbrella review of the evidence on the effectiveness of perioperative magnesium as an adjuvant for postoperative analgesia.

## 5. Conclusions

While most of included studies demonstrated a statistically significant improvement in outcomes related to postoperative pain when systemic magnesium was administered during the perioperative period, current findings reveal that our confidence in the beneficial effect of perioperative magnesium on postoperative pain is limited. While the updated meta-analysis with TSA suggested that the existing evidence is sufficient, additional research is needed to confirm the objective efficacy of perioperative magnesium for postoperative analgesia as well as research with reliable assessment of potential biases and appropriate interpretation of heterogeneity. Further studies applying standardized definitions of outcomes and magnesium administration protocols for evaluating postoperative pain could present more exact estimates, decrease the risk of heterogeneity, and increase reliability.

## Figures and Tables

**Table 1:** Study characteristics in adult and pediatric patients.

Patients Group	First Author (Year)	Search Period	Type of Anesthesia	Route of Magnesium Administration	Number of Included RCT	Number of Participant (Magnesium/Control)	Type of Surgery
Adult patients	Shi (2021)	October, 2020	GA RA	IA	11	677 (343/334)	Arthroscopic knee surgery
	Ma (2021)	February, 2020	GA RA	IV, IT, ED, local	8	880 (440/440)	Cesarean section
	Wang (2020)	March, 2020	RA	IT	10	720 (360/360)	Surgery procedure
	Li (2020)	October, 2019	RA GA + RA	ED	11	724 (362/362)	Surgical procedure
	Ng (2020)	January, 2019	GA RA	IV	51	3311	Non-cardiac surgery
	Chen (2018)	June, 2018	GA	IV	4	263 (131/132)	Laparoscopic cholecystectomy
	Wang (2017)	November, 2016	RA	IT, ED	9	827	Cesarean section
	Zeng (2016)	January, 2016	GA	IA	8	513 (242/271)	Arthroscopic surgery
	Guo (2015)	September, 2014	GA RA	IV	27	1504	Surgical procedure
	De Oliveira (2013)	June, 2012	GA	IV	20	1257 (639/618)	Surgical procedure
	Albrecht (2013)	January, 2012	GA RA	IV	25	1461 (731/730)	Surgical procedure
	Pascual-Ramirez (2013)	December, 2011	RA	IT	12	817 (412/405)	Below-umbilicus procedure
	Murphy (2013)	July, 2011	GA RA	IV	22	1177 (599/578)	Surgery procedure
Pediatric patients	Kawakami (2018)	November, 2017	RA	ED	6	371 (179/192)	Surgical procedure
	Xie (2017)	June, 2016	GA	IV, local	10	665 (333/332)	Tonsillectomy
	Cho (2017)	January, 2017	GA	IV, local	10	655 (328/327)	Tonsillectomy

RCT, randomized controlled trial; GA, general anesthesia; RA, regional anesthesia; IA, intra-articular; IV, intravenous; IT, intrathecal; ED, epidural.

**Table 2:** Summary of evidence: pain score in adult patients.

First Author, Year	Outcome	Study N	Participant N (Mg/Control)	MD, SMD, ES (95%CI)	Heterogeneity	Quality of Evidence (GRADE)
Shi 2021	At rest					
	2 h	8	423 (212/211)	MD −0.74 (−0.84, −0.64)	I^2^ = 0%, *p* = 0.51	Low
	4 h	6	303 (152/151)	MD −0.24 (−0.37, −0.11)	I^2^ = 0%, *p* = 0.51	Moderate
	12 h	6	304 (152/152)	MD −0.53 (−0.64, −0.41)	I^2^ = 0%, *p* = 0.51	High
	24 h	7	372 (186/186)	MD −0.33 (−0.42, −0.24)	I^2^ = 0%, *p* = 0.51	High
	At movement					
	2 h	7	279 (140/139)	MD −0.46 (−0.64, −0.27)	I^2^ = 0%, *p* = 0.51	High
	4 h	6	299 (150/149)	MD −0.85 (−1.40, −0.30)	I^2^ = 0%, *p* = 0.51	Moderate
	12 h	6	299 (150/149)	MD −0.83 (−1.17, −0.48)	I^2^ = 0%, *p* = 0.51	Moderate
	24 h	7	339 (170/169)	MD −0.58 (−0.79, −0.36)	I^2^ = 0%, *p* = 0.51	High
Ma 2021	Highest VAS	8	880 (440/440)	MD −0.74 (−1.03, −0.46)	I^2^ = 91.7%, *p* < 0.001	Low
	Last VAS	8	880 (440/440)	MD −0.47 (−0.71, −0.23)	I^2^ = 95.0%, *p* < 0.001	
Ng 2020	24 h	18	1232	MD −0.3 (−0.69, 0.09)	I^2^ = 91%	Low
Chen 2018	2 h	2	143 (71/72)	MD −0.45 (−0.88, −0.02)	I^2^ = 38%, *p* = 0.20	Low
	8 h	2	143 (71/72)	MD −0.62 (−0.95, −0.28)	I^2^ = 0%, *p* = 0.69	
	24 h	2	100 (50/50)	MD −0.38 (−0.79, 0.02)	I^2^ = 4%, *p* = 0.31	
Wang 2017	At rest	3	325 (164/161)	ES −1.206 (−2.084, −0.329)	I^2^ = 92.409, *p* < 0.001	Low
	At movement	2	265 (134/131)	ES −1.435 (−2.631, −0.240)	I^2^ = 94.265, *p* < 0.001	
Zeng 2016	Mg vs. placebo					Low
	24 or 48 h	5	289 (145/144)	MD −0.41 (−0.78, −0.05)	I^2^ = 80%, *p* = 0.0006	
	Mg vs. bupi					
	24 or 48 h	3	154 (77/77)	MD 0.17 (−0.92, 1.26)	I^2^ = 88%, *p* = 0.0002	
	Mg + bupi vs. bupi					
	18 or 24 h	3	154 (77/77)	MD −0.41 (−0.87, 0.04)	I^2^ = 73%, *p* = 0.03	
Guo 2015	At rest	NR	NR			CE
	total			SMD −1.43 (−2.74, −0.12)	*p* < 0.01	
	At movement					
	24 h			SMD −0.05 (−0.43, 0.32)	NR	
De Oliveira 2013	At rest					Moderate
	Early (0–4 h)	18	1153(567/586)	MD −0.74 (−1.08, −0.48)	I^2^ = 87%	
	Late (24 h)	13	606 (302/304)	MD −0.36 (−0.63, −0.09)	I^2^ = 71%	
	At movement					
	Early (0–4 h)	6	466 (224/242)	MD 0.52 (−1.15, 0.10)	I^2^ = 57%	
	Late (24 h)	5	285 (142/143)	MD −0.73 (−1.37, −0.1)	I^2^ = 72%	
Albrecht 2013	At rest					Low
	Early	15	868 (433/435)	MD −6.9 (−9.6, −4.2)	I^2^ = 79%, *p* < 0.00001	
	24 h	14	900 (434/466)	MD −4.2 (−6.3, −2.1)	I^2^ = 78%, *p* < 0.00001	
	At movement					
	Early	5	381 (190/191)	MD −6.5 (−10.0, −2.9)	I^2^ = 78%, *p* = 0.19	
	24 h	5	225 (112/113)	MD −9.2 (−16.1, −2.3)	I^2^ = 86%, *p* < 0.00001	
Murphy 2013	4–6 h	16	956 (477/479)	MD −0.67 (−1.12, −0.23)	I^2^ = 96%, *p* < 0.00001	Low
	20–24 h	15	908 (458/458)	MD −0.25 (−0.62, 0.71)	I^2^ = 94%, *p* < 0.00001	

N, number; VAS, visual analogue scale; bupi, bupivacaine; NR, not reported; CE, can’t evaluate; Mg, magnesium group; MD, mean difference; SMD, standardized mean difference; ES, effect size; CI, confidence interval.

**Table 3:** Summary of evidence: analgesic consumption (mg) in adult patients.

First Author, Year	Study Number	Participants Number (Mg/Control)	MD, SMD, ES (95%CI)	Heterogeneity	Quality of Evidence (GRADE)
Shi 2021	8	449 (229/220)	MD −4.23 (−4.64, −3.82)	I^2^ = 27%, *p* = 0.21	High
Ma 2021	5	290 (145/145)	SMD −3.20 (−5.45, −0.95)	I^2^ = 97.6%, *p* < 0.001	Very low
Li 2020	5	300 (150/150)	SMD −2.65 (−4.23, −1.06)	I^2^ = 96%, *p* < 0.00001	Very low
Ng 2020	19	945	MD −5.41 (−7.08, −3.74)	I^2^ = 92%, *p* < 0.001	Low
Chen 2018	2	143 (71/72)	SMD −0.40 (−0.73, −0.07)	I^2^ = 0%, *p* = 0.57	Moderate
Wang 2017	4	193/193	ES −1.620 (−2.434, −0.806)	I^2^ = 83.166%, *p* < 0.001	Low
Guo 2015	NR	NR	SMD −1.72 (−3.21, −0.23)	NR	CE
De Oliveira 2013	16	921 (479/442)	MD −10.52 (−13.50, −7.54)	I^2^ = 88%	Low
Albrecht 2013	19	1054 (527/527)	MD −7.6 (−9.5, −5.8)	I^2^ = 92%, *p* < 0.00001	Low
Murphy 2013	12	698 (349/349)	MD −7.40 (−9.40, −5.41)	I^2^ = 87%, *p* < 0.00001	Low

Mg, magnesium group; MD, mean difference; SMD, standardized mean difference; ES, effect size; CI, confidence interval; NR, not reported; CE, cannot evaluate.

**Table 4:** Summary of evidence: time to first analgesic request (min) in adult patients.

First Author, Year	Study Number	Participants Number (Mg/Control)	MD, SMD, RoM (95%CI)	Heterogeneity	Quality of Evidence (GRADE)
Shi 2021	11	613 (311/302)	MD, 329.99 (228.73,431.24)	I^2^ = 99%, *p* < 0.00001	Low
Ma 2021	8	880 (440/440)	SMD, −3.0. (−4.32, −1.74)	I^2^ = 96.3%, *p* < 0.001	Low
Li 2020	6	400 (200/200)	SMD, 4.96 (2.75, 7.17)	I^2^ = 98%, *p* < 0.00001	Very low
Ng 2020	11	824	MD, 143 (103, 183)	I^2^ = 99%, *p* < 0.001	Low
Wang 2020	9	660 (330/330)	RoM, 1.23 (1.13, 1.33)	I^2^ = 96%, *p* < 0.00001	Low
Zeng 2016	4 (Mg vs. placebo)	229 (115/114)	MD, 3.59 (0.26, 6.93)	I^2^ = 99%, *p* < 0.00001	Low
	3 (Mg vs. bupi)	154 (77/77)	MD, −0.82 (−5.83, 4.20)	I^2^ = 99%, *p* < 0.00001	
	3 (Mg + bupi vs. bupi)	154 (77/77)	MD, 6.25 (5.22, 7.29)	I^2^ = 69%, *p* = 0.04	
De Oliveira 2013	4	339 (161/178)	MD, 4.4 (−6.9, 15.9)	NR	CE
Albrecht 2013	4	298 (149/149)	MD, 7.2 (−1.9, 16.2)	I^2^ = 90%, *p* < 0.00001	Low
Pascual-Ramirez 2013	10	NR	MD, 85 SMD, 0.98 (0.51, 1.37)	I^2^ = 56%, *p* < 0.001	Moderate

Mg, magnesium group; Bupi, bupivacaine; MD, mean difference; SMD, standardized mean difference; RoM, ratio of means; CI, confidence interval; NR, not reported; CE, can’t evaluate.

**Table 5:** Summary of evidence in pediatric patients.

First Author, Year	Outcome	Study N	Participant N (Mg/Control)	RR, SMD, MD (95%CI)	Heterogeneity	Quality of Evidence (GRADE)
Kawakami 2018	Incidence of rescue analgesia	4	247 (117/130)	RR 0.45 (0.24, 0.86)	I^2^ = 62.5%, *p* = 0.046	Very low
Cho 2017	Pain score					
	15 min	6	405 (203/202)	SMD −0.26 (−0.52, 0.00)	I^2^ = 40.36%, *p* = 0.1232	Low
	1 h	9	615 (308/307)	SMD 0.05 (−0.70, 0.80)	I^2^ = 94.94%, *p* < 0.0001	
	24 h	6	330 (165/165)	SMD −0.39 (−0.71, −0.07)	I^2^ = 50.56%, *p* = 0.0727	
Xie 2017	Pain score					
	(mCHEOPs)					Low
	15 min	2	160 (80/80)	MD 0.17 (−0.02, 0.35)	I^2^ = 0%, *p* = 0.77	
	1 h	2	160 (80/80)	MD −0.59 (−3.11, 1.93)	I^2^ = 98%, *p* < 0.00001	
	Incidence of rescue analgesia	5	305 (153/152)	RR 0.53 (0.31, 0.91)	I^2^ = 69%, *p* = 0.01	Low

N, number; RR, risk ratio; SMD, standardized mean difference; MD, mean difference; CI, confidence interval; mCHEOPs, (modified-Children’s Hospital of Eastern Ontario Pain scale).

**Table 6:** Pain score results of updated meta-analysis and TSA in adult patients.

Postoperative Pain Score		Quantitative Meta-Analysis (SMD; 95% CI; *p*_chi_^2^; I^2^)	Description of Trial Sequential Analysis (TSA)
PACU	Rest (0 h)	0.395; 0.178–0.612; <0.001; 85.9%	Pain at rest (0 h): TSA indicated that 98.7% (2487 of 2520 patients) of the RIS was accrued. The cumulative *Z* curve crossed both the conventional test boundary and the trial sequential monitoring boundary.
	Rest (0–1 h)	0.459; 0.229–0.689; <0.001; 87.1%	
	Movement (0 h)	0.437; −0.113–0.988; <0.001; 88.3%	Pain at movement (0 h): The trial sequential monitoring boundary was ignored due to too little information use. The cumulative *Z* curve did not cross the conventional test boundary.
	Movement (0–1 h)	0.485; −0.275–1.245; <0.001; 89.5%	
Early phase	Rest (4 h)	0.872; 0.638–1.106; <0.001; 91.2%	Pain at rest (4 h): TSA indicated that accrued number of patients (3830) exceed the RIS (2959). The cumulative *Z* curve crossed both the conventional test boundary and the trial sequential monitoring boundary.
	Rest (0–4 h)	0.705; 0.494–0.916; <0.001; 87.7%	
	Movement (4 h)	0.942; 0.364–1.520; <0.001; 93.2%	Pain at movement (4 h): TSA indicated that 89.0% (832 of 934 patients) of the RIS was accrued. The cumulative *Z* curve crossed both the conventional test boundary and the trial sequential monitoring boundary.
	Movement (0–4 h)	1.059; 0.561–1.556; <0.001; 89.6%	
Late phase	Rest (24 h)	0.470; 0.307–0.633; <0.001; 81.6%	Pain at rest (24 h): TSA indicated that accrued number of patients (3500) exceed the RIS (3115). The cumulative *Z* curve crossed both the conventional test boundary and the trial sequential monitoring boundary.
	Movement (24 h)	0.679; 0.388–0.970; <0.001; 61.1%	Pain at movement (24 h): TSA indicated that only 60.8% (507 of 834 patients) of the RIS was accrued. The cumulative *Z* curve crossed both the conventional test boundary and the trial sequential monitoring boundary.

PACU, post-anesthesia care unit; SMD, standardized mean difference; RIS, required information size.

**Table 7:** Pain-related outcomes of updated meta-analysis and TSA in pediatric patients.

Postoperative Outcomes			Quantitative Meta-Analysis (SMD or RR; 95% CI; *p*_chi_^2^; I^2^)	Description of Trial Sequential Analysis (TSA)
Pain score	PACU	0 h	0.811; 0.194–1.429; <0.001; 94.2%	Pain (0 h): TSA indicated that only 12.6% (853 of 6776 patients) of the RIS was accrued. The cumulative *Z* curve crossed the conventional test boundary but returned within the conventional boundary during TSA.
		0–1 h	0.553; 0.065–1.040; <0.001; 90.7%	
	Early phase	4 h	0.536; 0.064–1.008; <0.001; 82.4%	Pain (4 h): The trial sequential monitoring boundary was ignored due to too little information use. The cumulative *Z* curve crossed the conventional test boundary but did not cross the trial sequential monitoring boundary.
		0–4 h	0.452; −0.010–0.914; <0.001; 89.7%	
	Late phase	24 h	0.342; −0.360–1.044; <0.001; 93.8%	Pain (24 h): The trial sequential monitoring boundary was ignored due to too little information use. The cumulative *Z* curve did not cross the conventional test boundary.
Time to first analgesic			−1.222; −2.345–0.098; <0.001; 92.4%	The trial sequential monitoring boundary was ignored due to too little information use. The cumulative *Z* curve crossed the conventional test boundary but did not cross the trial sequential monitoring boundary.
Analgesic consumption			1.144; 0.370–1.917; <0.001; 88.8%	TSA indicated that only 10.1% (292 of 2881 patients) of the RIS was accrued. The cumulative *Z* curve crossed the conventional test boundary but did not cross the trial sequential monitoring boundary.
Incidence of rescue analgesic			1.991 *; 1.385–2.862; 0.014; 58.2%	TSA indicated that only 80.8% (552 of 683 patients) of the RIS was accrued. The cumulative *Z* curve crossed both the conventional test boundary and the trial sequential monitoring boundary.

PACU, post-anesthesia care unit; SMD, standardized mean difference; RR, risk ratio; CI, confidence interval; RIS, required information size. *, RR.

## Data Availability

The datasets used and analyzed during the current study are available from the corresponding author upon reasonable request.

## Supplementary Materials

The following are available online at https://www.mdpi.com/article/10.3390/jpm11121273/s1, Figure S1: Flow diagram of updated meta-analysis; Figure S2: Forest plot showing resting pain score at postoperative 0 h in adult patients; Figure S3: Forest plot showing resting pain score at postoperative 0–1 h in adult patients; Figure S4: Forest plot showing resting pain score at postoperative 0–4 h in adult patients; Figure S5: Forest plot showing resting pain score at postoperative 24 h in adult patients; Figure S6: Forest plot showing dynamic pain score at postoperative 0 h in adult patients; Figure S7: Forest plot showing dynamic pain score at postoperative 0–1 h in adult patients; Figure S8: Forest plot showing dynamic pain score at postoperative 4 h in adult patients; Figure S9: Forest plot showing dynamic pain score at postoperative 0–4 h in adult patients; Figure S10: Forest plot showing dynamic pain score at postoperative 24 h in adult patients; Figure S11: Forest plot showing pain score at postoperative 0 h in pediatric patients; Figure S12: Forest plot showing pain score at postoperative 0–1 h in pediatric patients; Figure S13: Forest plot showing pain score at postoperative 4 h in pediatric patients; Figure S14: Forest plot showing pain score at postoperative 0–4 h in pediatric patients; Figure S15: Forest plot showing pain score at postoperative 24 h in pediatric patients; Figure S16: Forest plot showing time to first analgesic in pediatric patients; Figure S17: Forest plot showing analgesic consumption in pediatric patients; Figure S18: TSA for resting pain score at postoperative 0 h in adult patients; Figure S19: TSA for resting pain score at postoperative 24 h in adult patients; Figure S20: TSA for dynamic pain score at postoperative 0 h in adult patients; Figure S21: TSA for dynamic pain score at postoperative 4 h in adult patients; Figure S22: TSA for dynamic pain score at postoperative 24 h in adult patients; Figure S23: TSA for pain score at postoperative 0 h in pediatric patients; Figure S24: TSA for pain score at postoperative 4 h in pediatric patients; Figure S25: TSA for pain score at postoperative 24 h in pediatric patients; Figure S26: TSA for the time to first analgesic in pediatric patients; Figure S27: TSA for the analgesic consumption in pediatric patients; Figure S28: TSA for the incidence of rescue analgesic in pediatric patients; Figure S29: Funnel plot for resting pain score at postoperative 0 h in adult patients; Figure S30: Funnel plot for resting pain score at postoperative 0–1 h in adult patients; Figure S31: Funnel plot for resting pain score at postoperative 4 h in adult patients; Figure S32: Funnel plot for resting pain score at postoperative 0–4 h in adult patients; Figure S33: Funnel plot for resting pain score at postoperative 24 h in adult patients; Figure S34: Funnel plot for dynamic pain score at postoperative 0 h in adult patients; Figure S35: Funnel plot for dynamic pain score at postoperative 0–1 h in adult patients; Figure S36: Funnel plot for dynamic pain score at postoperative 4 h in adult patients; Figure S37: Funnel plot for dynamic pain score at postoperative 0–4 h in adult patients; Figure S38: Funnel plot for dynamic pain score at postoperative 24 h in adult patients; Figure S39: Funnel plot for the time to first analgesic in adult patients; Figure S40: Funnel plot for the analgesic consumption in adult patients; Figure S41: Funnel plot for pain score at postoperative 0 h in pediatric patients; Figure S42: Funnel plot for pain score at postoperative 0–1 h in pediatric patients; Figure S43: Funnel plot for pain score at postoperative 4 h in pediatric patients; Figure S44: Funnel plot for pain score at postoperative 0–4 h in pediatric patients; Figure S45: Funnel plot for pain score at postoperative 24 h in pediatric patients; File S1: PRISMA checklist; File S2: search term of umbrella review; File S3: search term of updated meta-analysis
