# Supplementary material for: Perioperative Magnesium for Postoperative Analgesia: An Umbrella Review of Systematic Reviews and Updated Meta-Analysis of Randomized Controlled Trials
Source: J Pers Med. 2021 Dec 2;11(12):1273. doi: 10.3390/jpm11121273 (PMC8708823; doi:10.3390/jpm11121273)
Supplement: Supplementary file 1 [file jpm-11-01273-s001.zip › Supplementary figure legend.pdf]

Figure S1 PRISMA flow diagram of updated meta-analysis

Figure S2 Forest plot showing resting pain score at postoperative 0 h in adult patients. The figure depicts individual trials as filled squares with relative sample size and the 95% confidence interval (CI) of the difference as a solid line. The diamond shape indicates the pooled estimate and uncertainty for the combined effect.

Figure S3 Forest plot showing resting pain score at postoperative 0-1 h in adult patients. The figure depicts individual trials as filled squares with relative sample size and the 95% confidence interval (CI) of the difference as a solid line. The diamond shape indicates the pooled estimate and uncertainty for the combined effect.

Figure S4 Forest plot showing resting pain score at postoperative 0-4 h in adult patients. The figure depicts individual trials as filled squares with relative sample size and the 95% confidence interval (CI) of the difference as a solid line. The diamond shape indicates the pooled estimate and uncertainty for the combined effect.

Figure S5 Forest plot showing resting pain score at postoperative 24 h in adult patients. The figure depicts individual trials as filled squares with relative sample size and the 95% confidence interval (CI) of the difference as a solid line. The diamond shape indicates the pooled estimate and uncertainty for the combined effect.

Figure S6 Forest plot showing dynamic pain score at postoperative 0 h in adult patients. The figure depicts individual trials as filled squares with relative sample size and the 95% confidence interval (CI) of the difference as a solid line. The diamond shape indicates the pooled estimate and uncertainty for the combined effect.

Figure S7 Forest plot showing dynamic pain score at postoperative 0-1 h in adult patients. The figure depicts individual trials as filled squares with relative sample size and the 95% confidence interval (CI) of the difference as a solid line. The diamond shape indicates the pooled estimate and uncertainty for the combined effect.

Figure S8 Forest plot showing dynamic pain score at postoperative 4 h in adult patients. The figure depicts individual trials as filled squares with relative sample size and the 95% confidence interval (CI) of the difference as a solid line. The diamond shape indicates the pooled estimate and uncertainty for the combined effect.

Figure S9 Forest plot showing dynamic pain score at postoperative 0-4 h in adult patients. The figure depicts individual trials as filled squares with relative sample size and the 95% confidence interval (CI) of the difference as a solid line. The diamond shape indicates the pooled estimate and uncertainty for the combined effect.

Figure S10 Forest plot showing dynamic pain score at postoperative 24 h in adult patients. The figure depicts individual trials as filled squares with relative sample size and the 95% confidence interval (CI) of the difference as a solid line. The diamond shape indicates the pooled estimate and uncertainty for the combined effect.

Figure S11 Forest plot showing pain score at postoperative 0 h in pediatric patients. The figure depicts individual trials as filled squares with relative sample size and the 95% confidence interval (CI) of the difference as a solid line. The diamond shape indicates the pooled estimate and uncertainty for the combined effect.

Figure S12 Forest plot showing pain score at postoperative 0-1 h in pediatric patients. The figure depicts individual trials as filled squares with relative sample size and the 95% confidence interval (CI) of the difference as a solid line. The diamond shape indicates the pooled estimate and uncertainty for the combined effect.

Figure S13 Forest plot showing pain score at postoperative 4 h in pediatric patients. The figure depicts individual trials as filled squares with relative sample size and the 95% confidence interval (CI) of the difference as a solid line. The diamond shape indicates the pooled estimate and uncertainty for the combined effect.

Figure S14 Forest plot showing pain score at postoperative 0-4 h in pediatric patients. The figure depicts individual trials as filled squares with relative sample size and the 95% confidence interval (CI) of the difference as a solid line. The diamond shape indicates the pooled estimate and uncertainty for the combined effect.

Figure S15 Forest plot showing pain score at postoperative 24 h in pediatric patients. The figure depicts individual trials as filled squares with relative sample size and the 95% confidence interval (CI) of the difference as a solid line. The diamond shape indicates the pooled estimate and uncertainty for the combined effect.

Figure S16 Forest plot showing time to first analgesic in pediatric patients. The figure depicts individual trials as filled squares with relative sample size and the 95% confidence interval (CI) of the difference as a solid line. The diamond shape indicates the pooled estimate and uncertainty for the combined effect.

Figure S17 Forest plot showing analgesic consumption in pediatric patients. The figure depicts individual trials as filled squares with relative sample size and the 95% confidence interval (CI) of the difference as a solid line. The diamond shape indicates the pooled estimate and uncertainty for the combined effect.

Figure S18 TSA for resting pain score at postoperative 0 h in adult patients**.** Complete blue line represents the cumulative Z curve, etched red line represents conventional test boundary, complete outer red line represents the trial sequential monitoring boundary, and complete inner red line represents futility boundary. The cumulative *Z* curve crossed the conventional test boundary and the trial sequential monitoring boundary.

Figure S19 TSA for resting pain score at postoperative 24 h in adult patients**.** Complete blue line represents the cumulative Z curve, etched red line represents conventional test boundary, complete outer red line represents the trial sequential monitoring boundary, and complete

inner red line represents futility boundary. The cumulative *Z* curve crossed the conventional test boundary and the trial sequential monitoring boundary.

Figure S20 TSA for dynamic pain score at postoperative 0 h in adult patients**.** Complete blue line represents the cumulative Z curve, etched red line represents conventional test boundary. Trial sequential monitoring boundary is ignored due to too little information use. The cumulative *Z* curve did not cross the conventional boundary.

Figure S21 TSA for dynamic pain score at postoperative 4 h in adult patients**.** Complete blue line represents the cumulative Z curve, etched red line represents conventional test boundary, complete outer red line represents the trial sequential monitoring boundary, and complete inner red line represents futility boundary. The cumulative *Z* curve crossed the conventional test boundary and the trial sequential monitoring boundary.

Figure S22 TSA for dynamic pain score at postoperative 24 h in adult patients**.** Complete blue line represents the cumulative Z curve, etched red line represents conventional test boundary, complete outer red line represents the trial sequential monitoring boundary, and complete inner red line represents futility boundary. The cumulative *Z* curve crossed the conventional test boundary and the trial sequential monitoring boundary.

Figure S23 TSA for pain score at postoperative 0 h in pediatric patients**.** Complete blue line represents the cumulative Z curve, etched red line represents conventional test boundary, and complete inner red line represents futility boundary. Trial sequential monitoring boundary is ignored due to too little information use. The cumulative *Z* curve did not cross the conventional boundary.

Figure S24 TSA for pain score at postoperative 4 h in pediatric patients**.** Complete blue line represents the cumulative Z curve, etched red line represents conventional test boundary, complete outer red line represents the trial sequential monitoring boundary, and complete inner red line represents futility boundary. The cumulative *Z* curve crossed the conventional test boundary but did not cross the trial sequential monitoring boundary.

Figure S25 TSA for pain score at postoperative 24 h in pediatric patients**.** Complete blue line represents the cumulative Z curve, etched red line represents conventional test boundary, complete outer red line represents the trial sequential monitoring boundary, and complete inner red line represents futility boundary. The cumulative *Z* curve did not cross the conventional boundary.

.

Figure S26 TSA for the time to first analgesic in pediatric patients**.** Complete blue line represents the cumulative Z curve, etched red line represents conventional test boundary, complete outer red line represents the trial sequential monitoring boundary, and complete inner red line represents futility boundary. The cumulative *Z* curve crossed the conventional test boundary but did not cross the trial sequential monitoring boundary.

Figure S27 TSA for the analgesic consumption in pediatric patients**.** Complete blue line represents the cumulative Z curve, etched red line represents conventional test boundary, complete outer red line represents the trial sequential monitoring boundary, and complete inner red line represents futility boundary. The cumulative *Z* curve crossed the conventional test boundary but did not cross the trial sequential monitoring boundary.

Figure S28 TSA for the incidence of rescue analgesic in pediatric patients**.** Complete blue line represents the cumulative Z curve, etched red line represents conventional test boundary, complete outer red line represents the trial sequential monitoring boundary, and complete

inner red line represents futility boundary. The cumulative *Z* curve crossed the conventional test boundary and the trial sequential monitoring boundary.

Figure S29 Funnel for resting pain score at postoperative 0 h in adult patients**.** White circles: comparisons included. Black circles: inputted comparisons using the trim-and-fill method. White diamond: pooled observed log risk ratio. Black diamond: pooled inputted log risk ratio.

Figure S30 Funnel for resting pain score at postoperative 0-1 h in adult patients**.** White circles: comparisons included. Black circles: inputted comparisons using the trim-and-fill method. White diamond: pooled observed log risk ratio. Black diamond: pooled inputted log risk ratio.

Figure S31 Funnel for resting pain score at postoperative 4 h in adult patients**.** White circles: comparisons included. Black circles: inputted comparisons using the trim-and-fill method. White diamond: pooled observed log risk ratio. Black diamond: pooled inputted log risk ratio.

Figure S32 Funnel for resting pain score at postoperative 0-4 h in adult patients**.** White circles: comparisons included. Black circles: inputted comparisons using the trim-and-fill method. White diamond: pooled observed log risk ratio. Black diamond: pooled inputted log risk ratio.

Figure S33 Funnel for resting pain score at postoperative 24 h in adult patients**.** White circles: comparisons included. Black circles: inputted comparisons using the trim-and-fill method. White diamond: pooled observed log risk ratio. Black diamond: pooled inputted log risk ratio.

Figure S34 Funnel for dynamic pain score at postoperative 0 h in adult patients**.** White circles: comparisons included. Black circles: inputted comparisons using the trim-and-fill method. White diamond: pooled observed log risk ratio. Black diamond: pooled inputted log risk ratio.

Figure S35 Funnel for dynamic pain score at postoperative 0-1 h in adult patients**.** White circles: comparisons included. Black circles: inputted comparisons using the trim-and-fill method. White diamond: pooled observed log risk ratio. Black diamond: pooled inputted log risk ratio.

Figure S36 Funnel for dynamic pain score at postoperative 4 h in adult patients**.** White circles: comparisons included. Black circles: inputted comparisons using the trim-and-fill method. White diamond: pooled observed log risk ratio. Black diamond: pooled inputted log risk ratio.

Figure S37 Funnel for dynamic pain score at postoperative 0-4 h in adult patients**.** White circles: comparisons included. Black circles: inputted comparisons using the trim-and-fill method. White diamond: pooled observed log risk ratio. Black diamond: pooled inputted log risk ratio.

Figure S38 Funnel for dynamic pain score at postoperative 24 h in adult patients**.** White circles: comparisons included. Black circles: inputted comparisons using the trim-and-fill method. White diamond: pooled observed log risk ratio. Black diamond: pooled inputted log risk ratio.

Figure S39 Funnel for the time to first analgesic in adult patients**.** White circles: comparisons included. Black circles: inputted comparisons using the trim-and-fill method. White diamond: pooled observed log risk ratio. Black diamond: pooled inputted log risk ratio.

Figure S40 Funnel for the analgesic consumption in adult patients**.** White circles: comparisons included. Black circles: inputted comparisons using the trim-and-fill method. White diamond: pooled observed log risk ratio. Black diamond: pooled inputted log risk ratio.

Figure S41 Funnel for pain score at postoperative 0 h in pediatric patients**.** White circles: comparisons included. Black circles: inputted comparisons using the trim-and-fill method. White diamond: pooled observed log risk ratio. Black diamond: pooled inputted log risk ratio.

Figure S42 Funnel for pain score at postoperative 0-1 h in pediatric patients**.** White circles: comparisons included. Black circles: inputted comparisons using the trim-and-fill method.

White diamond: pooled observed log risk ratio. Black diamond: pooled inputted log risk ratio.

Figure S43 Funnel for pain score at postoperative 4 h in pediatric patients**.** White circles: comparisons included. Black circles: inputted comparisons using the trim-and-fill method. White diamond: pooled observed log risk ratio. Black diamond: pooled inputted log risk ratio.

Figure S44 Funnel for pain score at postoperative 0-4 h in pediatric patients**.** White circles: comparisons included. Black circles: inputted comparisons using the trim-and-fill method. White diamond: pooled observed log risk ratio. Black diamond: pooled inputted log risk ratio.

Figure S45 Funnel for pain score at postoperative 24 h in pediatric patients**.** White circles: comparisons included. Black circles: inputted comparisons using the trim-and-fill method. White diamond: pooled observed log risk ratio. Black diamond: pooled inputted log risk ratio.
