# Supplementary material for: Perioperative Magnesium for Postoperative Analgesia: An Umbrella Review of Systematic Reviews and Updated Meta-Analysis of Randomized Controlled Trials
Source: J Pers Med. 2021 Dec 2;11(12):1273. doi: 10.3390/jpm11121273 (PMC8708823; doi:10.3390/jpm11121273)
Supplement: Supplementary file 1 [file jpm-11-01273-s001.zip › Supplementary file S2_search term_umbrella review.pdf]

Ovid-Medline

		Search term	Number of reference
Study design (SD)	1	exp systematic review/	171760
	2	systematic review.ab,ti.	161599
	3	systematic review.mp	191484
	4	systemic review.mp	1541
	5	exp meta-analysis as topic/	23378
	6	meta-analysis.ab,ti.	152301
	7	metaanalysis.ab,ti.	1568
	8	pooled analysis.mp.	8829
	9	or/1-8	283999
Population (P)	10	exp General Surgery/	39985
	11	Surg$.mp.	3090005
	12	Oper$.mp.	1174027
	13	perioper$.mp.	105530
	14	postoper$.mp.	839310
	15	preoper$.mp.	316620
	16	exp Anesthesia/	198460
	17	Anesth$.mp.	387485
	18	Anaesth$.mp.	100506
	19	or/10-18	4063331
Intervention (I)	20	exp magnesium/	68494
	21	magnesium.mp.	102523
	22	magnesium.mp.	51578
	23	Or/20-22	102523
SD and P and I	24	9 and 19 and 23	136

Ovid-embase

		Search term	Number of reference
Study design (SD)	#1	'systematic review'/exp OR 'systematic review'	405056
	#2	systematic AND review	482177
	#3	systemic AND review	183703
	#4	'meta analysis'/exp	229091
	#5	'meta analysis' OR 'metaanalysis'	338083
	#6	#1 OR #2 OR #3 OR #4 OR #5	790762
Population (P)	#7	'general surgery'/exp	19097
	#8	surg$:ti,ab	32235
	#9	operat$:ti,ab	52563
	#10	perioper$:ti,ab	109
	#11	postoper$:ti,ab	302
	#12	preoper$.ti,ab	333
	#13	'anesthesia'/exp	388885
	#14	anesth$:ti,ab	569
	#15	anaesth$ ti,ab	575
	#16	#7 OR #8 OR #9 OR #10 OR #11 OR #12 OR #13 OR #14 OR #15	491944
Intervention (I)	#17	'magnesium'/exp	96671
	#18	magnesium.ti,ab	76454
	#19	magnesium.ab,ti.	183617
	#20	#17 OR #18 OR #19	183617
SD and P and I	#21	#6 AND #16 AND #20	238
