# Supplementary material for: Perioperative Magnesium for Postoperative Analgesia: An Umbrella Review of Systematic Reviews and Updated Meta-Analysis of Randomized Controlled Trials
Source: J Pers Med. 2021 Dec 2;11(12):1273. doi: 10.3390/jpm11121273 (PMC8708823; doi:10.3390/jpm11121273)
Supplement: Supplementary file 1 [file jpm-11-01273-s001.zip › Supplementary file S3_search term_updated meta-analysis.pdf]

Ovid-Medline

		Search term	Number of reference
Study design (SD)	1	randomized controlled trial.pt	549294
	2	randomized controlled trial$.mp	736532
	3	controlled clinical trial.pt	94504
	4	controlled clinical trial$.mp	124427
	5	random allocation.mp	107627
	6	exp double-blind method/	168124
	7	double-blind.mp	195841
	8	exp single-blind method/	31145
	9	single-blind.mp	35747
	10	or/1-9	954619
	11	clinical trial.pt	531433
	12	clinical trial$.mp	1025453
	13	exp clinical trial/	916124
	14	(clin$ adj25 trial$).mp	1096343
	15	((singl$ or doubl$ or tripl$ or trebl$) adj25 (blind$ or mask$)).mp	241869
	16	random$.mp	1319531
	17	exp research design/	471005
	18	research design.mp	145864
	19	or/11-18	2151583
	20	10 or 19	2151583
	21	Case report.tw.	265861
	22	Letter.pt.	1107388
	23	Historical article.pt.	366349
	24	Review.pt.	2635723
	25	or/21-24	4292576
	26	20 not 25	1788967
Population (P)	27	exp General Surgery/	39985
	28	Surg$.mp.	3090005
	29	Oper$.mp.	1174027
	30	perioper$.mp.	105530
	31	postoper$.mp.	839310
	32	preoper$.mp.	316620
	33	exp Anesthesia/	198460
	34	Anesth$.mp.	387485
	35	Anaesth$.mp.	100506
	36	or/27-35	4063331
Intervention (I)	37	exp magnesium/	68494
	38	magnesium.mp.	102523
	39	magnesium.ab,ti.	51578
	40	Or/37-39	102523
SD and P and I	41	26 and 36 and 40	949

Ovid-embase

		Search term	Number of reference
Study design (SD)	#1	randomi?ed AND controlled AND trial$	1037348
	#2	'controlled clinical trial (topic)'/exp	222481
	#3	controlled:ti,ab AND clinical:ti,ab AND trials:ti,ab	119660
	#4	controlled AND clinical AND trial$	1389787
	#5	'randomization'/exp	92492
	#6	'random allocation'/exp	92492
	#7	random AND allocation	5696
	#8	'double blind':ti,ab	208353
	#9	'single blind':ti,ab	20072
	#10	#1 OR #2 OR #3 OR #4 OR #5 OR #6 OR #7 OR #8 OR #9	1646204
	#11	'clinical trial (topic)'/exp	372059
	#12	clinical AND trial$	2390191
	#13	random$	381481
	#14	rct	49833
	#15	#11 OR #12 OR #13 OR #14	2791568
	#16	#10 OR #15	2954870
	#17	'case study'/exp	81925
	#18	'case report'/exp	2759590
	#19	'abstract report'/exp	89796
	#20	'letter'/exp	1131275
	#21	#17 OR #18 OR #19 OR #20	3825854
	#22	#16 NOT #21	2840480
Population (P)	#23	'general surgery'/exp	19097
	#24	surg$:ti,ab	32235
	#25	operat$:ti,ab	52563
	#26	perioper$:ti,ab	109
	#27	postoper$:ti,ab	302
	#28	preoper$.ti,ab	333
	#29	'anesthesia'/exp	388885
	#30	anesth$:ti,ab	569
	#31	anaesth$ ti,ab	575
	#32	#23 OR #24 OR #25 OR #26 OR #27 OR #28 OR #29 OR #30 OR #31	491944
Intervention (I)	#33	'magnesium'/exp	96671
	#34	magnesium	76454
	#35	magnesium	183617
	#36	#33 OR #34 OR #35	183617
SD and P and I	#37	#22 AND #32 AND #36	779

Cochrane

	Search term	Number of reference
1	MeSH descriptor [General Surgery] explode all trees	362
2	MeSH descriptor [Anesthesia] explode all trees	20061
3	MeSH descriptor [Magnesium] explode all trees	1210
4	(#1 OR #2)AND #3	30
