# Supplementary figures and images for: Perioperative Magnesium for Postoperative Analgesia: An Umbrella Review of Systematic Reviews and Updated Meta-Analysis of Randomized Controlled Trials
Source: J Pers Med. 2021 Dec 2;11(12):1273. doi: 10.3390/jpm11121273 (PMC8708823; doi:10.3390/jpm11121273)

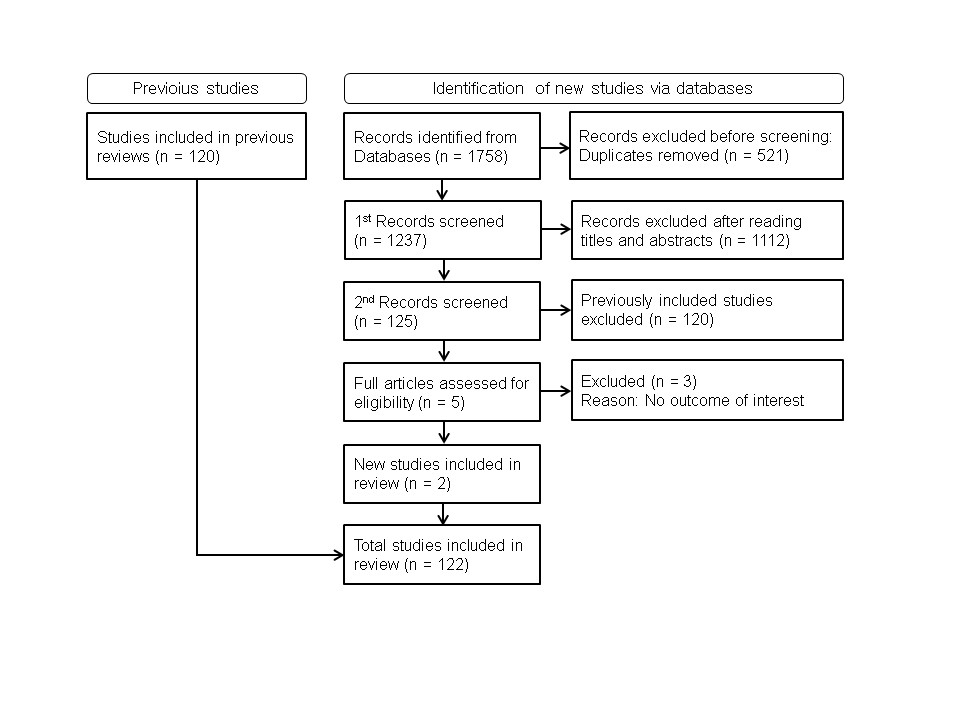

Supplement: Supplementary file 1 [file jpm-11-01273-s001.zip › S1.jpg]

# Alpha-spending Boundaries is a Two-sided graph

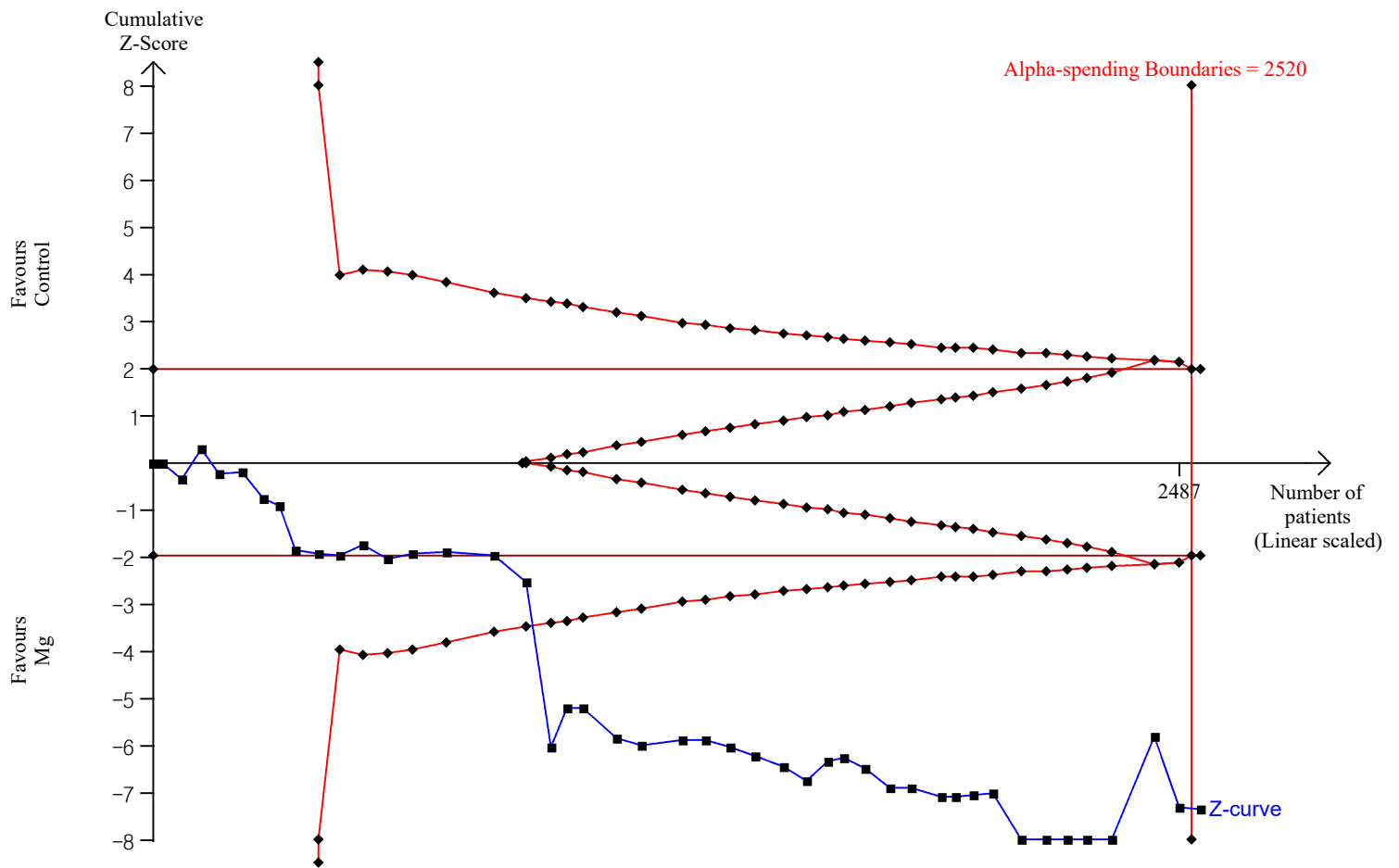

Supplement: Supplementary file 1 [file jpm-11-01273-s001.zip › S18.pdf]

Alpha-spending Boundaries is a Two-sided graph

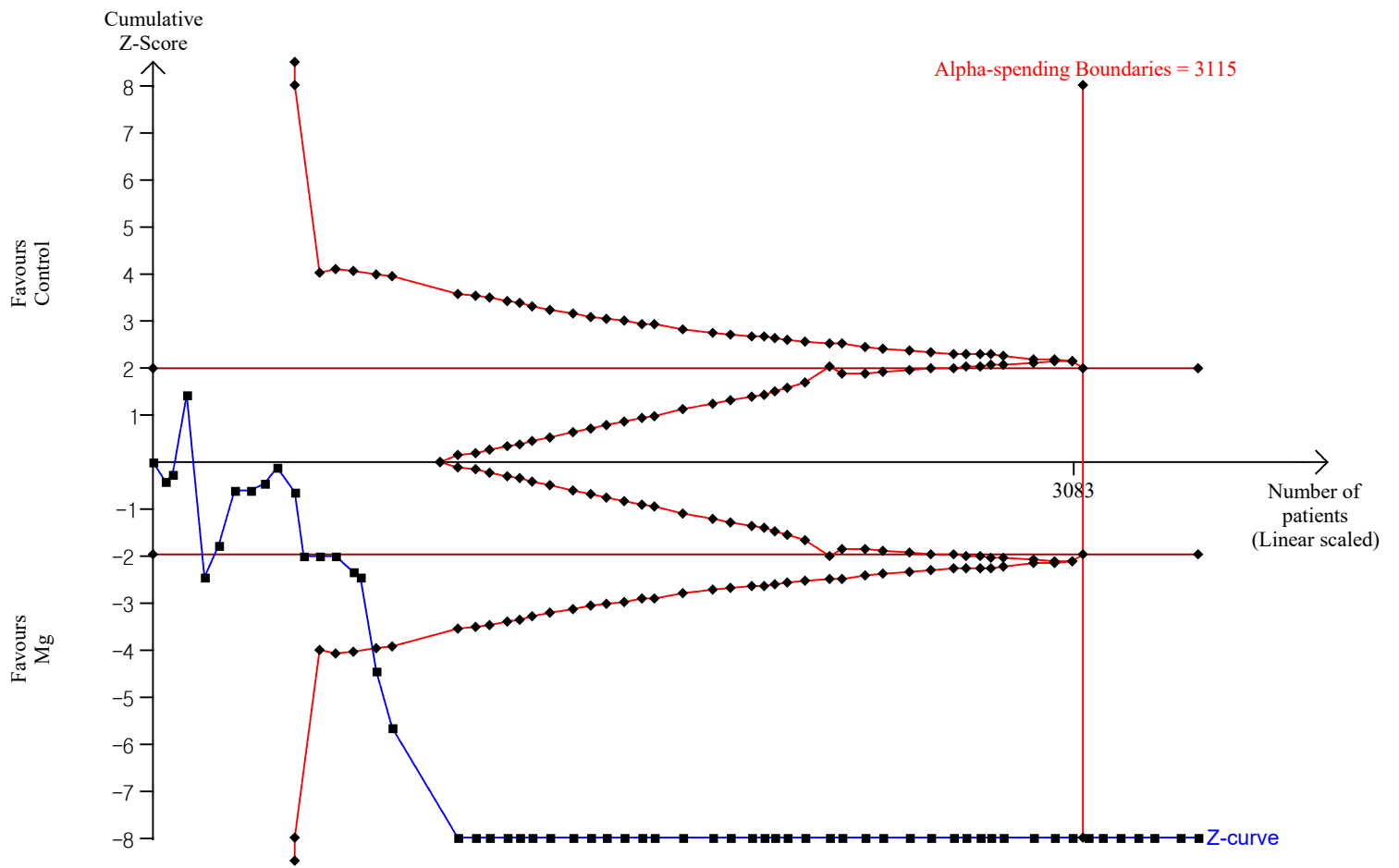

Supplement: Supplementary file 1 [file jpm-11-01273-s001.zip › S19.pdf]

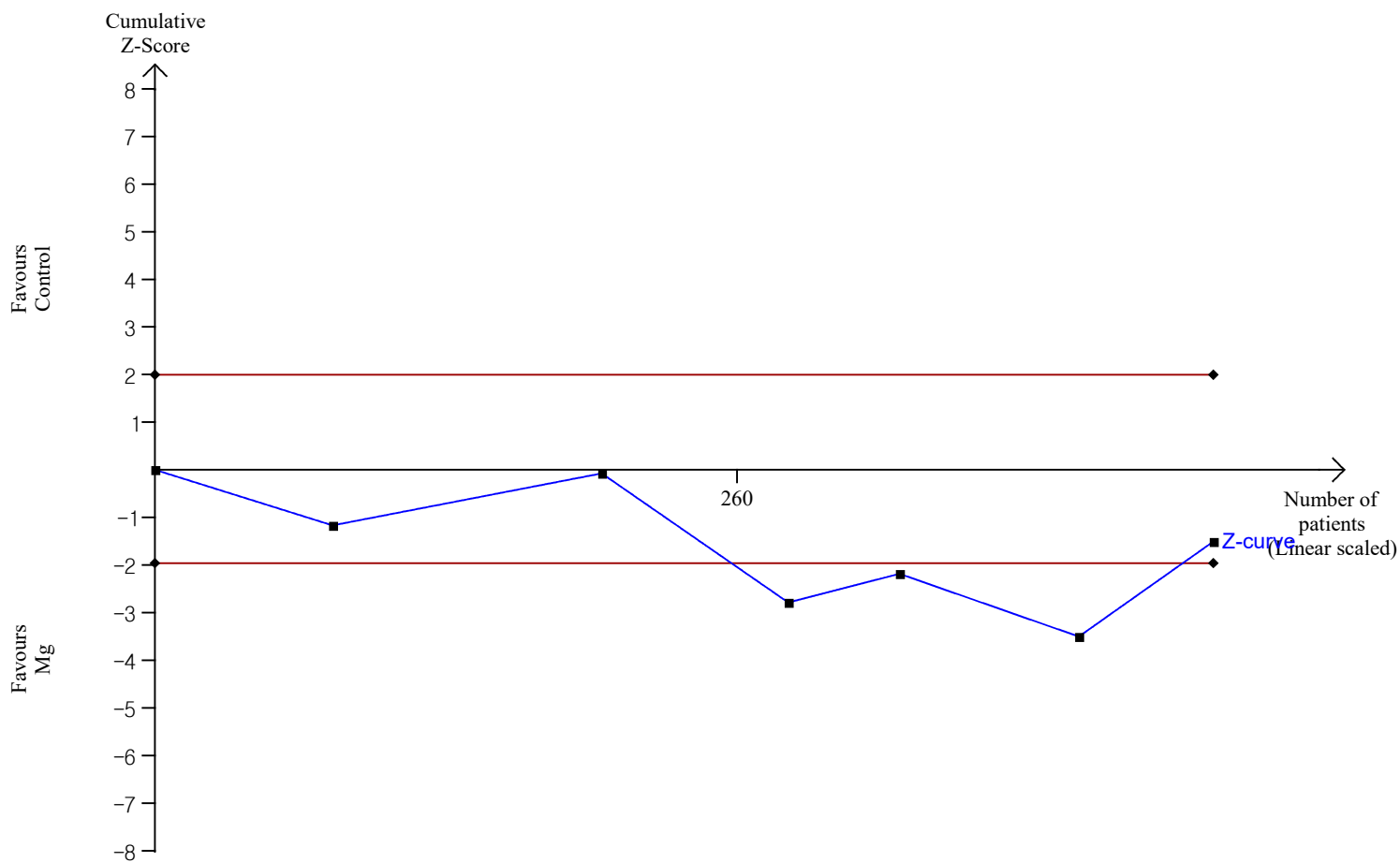

Supplement: Supplementary file 1 [file jpm-11-01273-s001.zip › S20.pdf]

# Alpha-spending Boundaries is a Two-sided graph

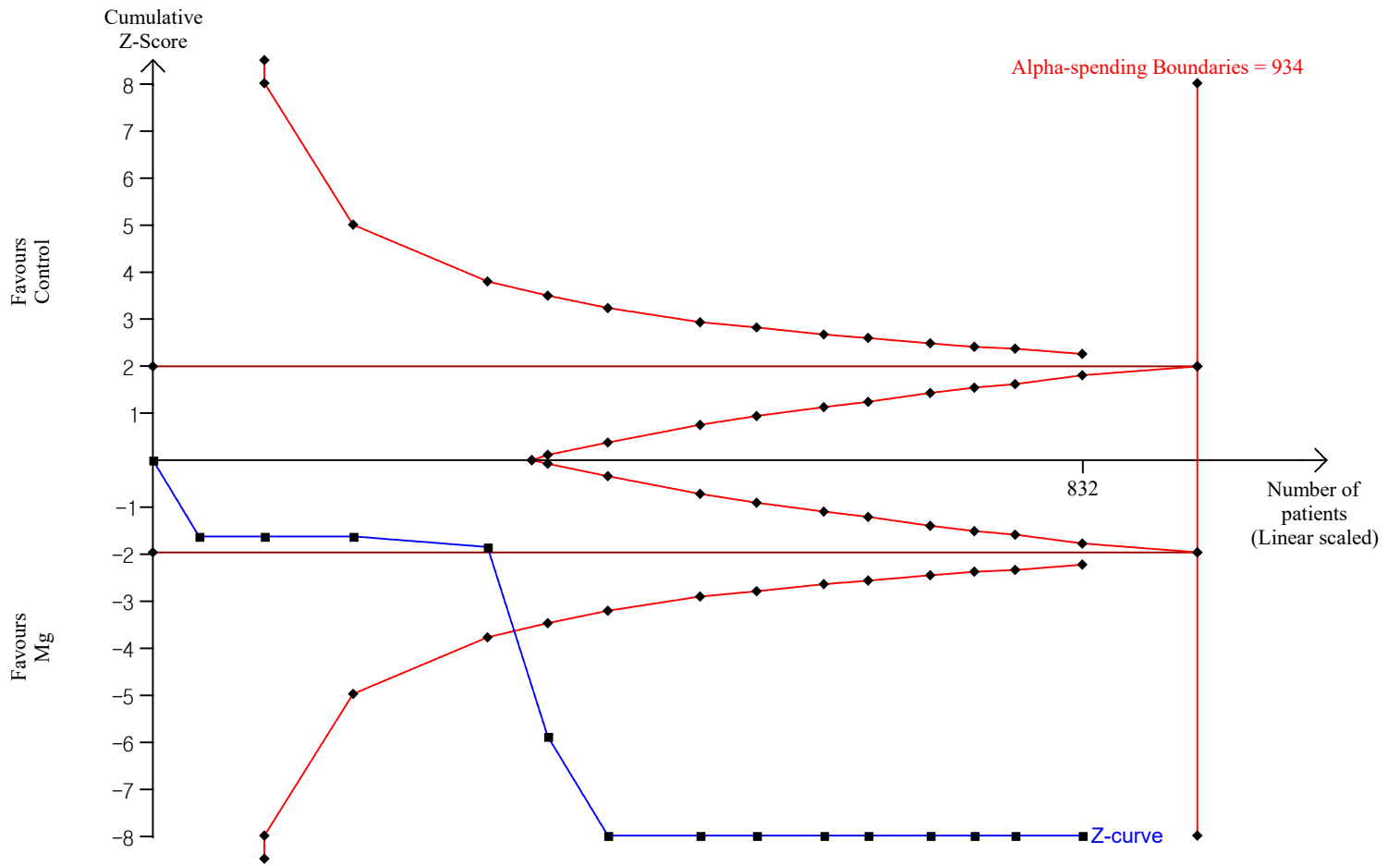

Supplement: Supplementary file 1 [file jpm-11-01273-s001.zip › S21.pdf]

# Alpha-spending Boundaries is a Two-sided graph

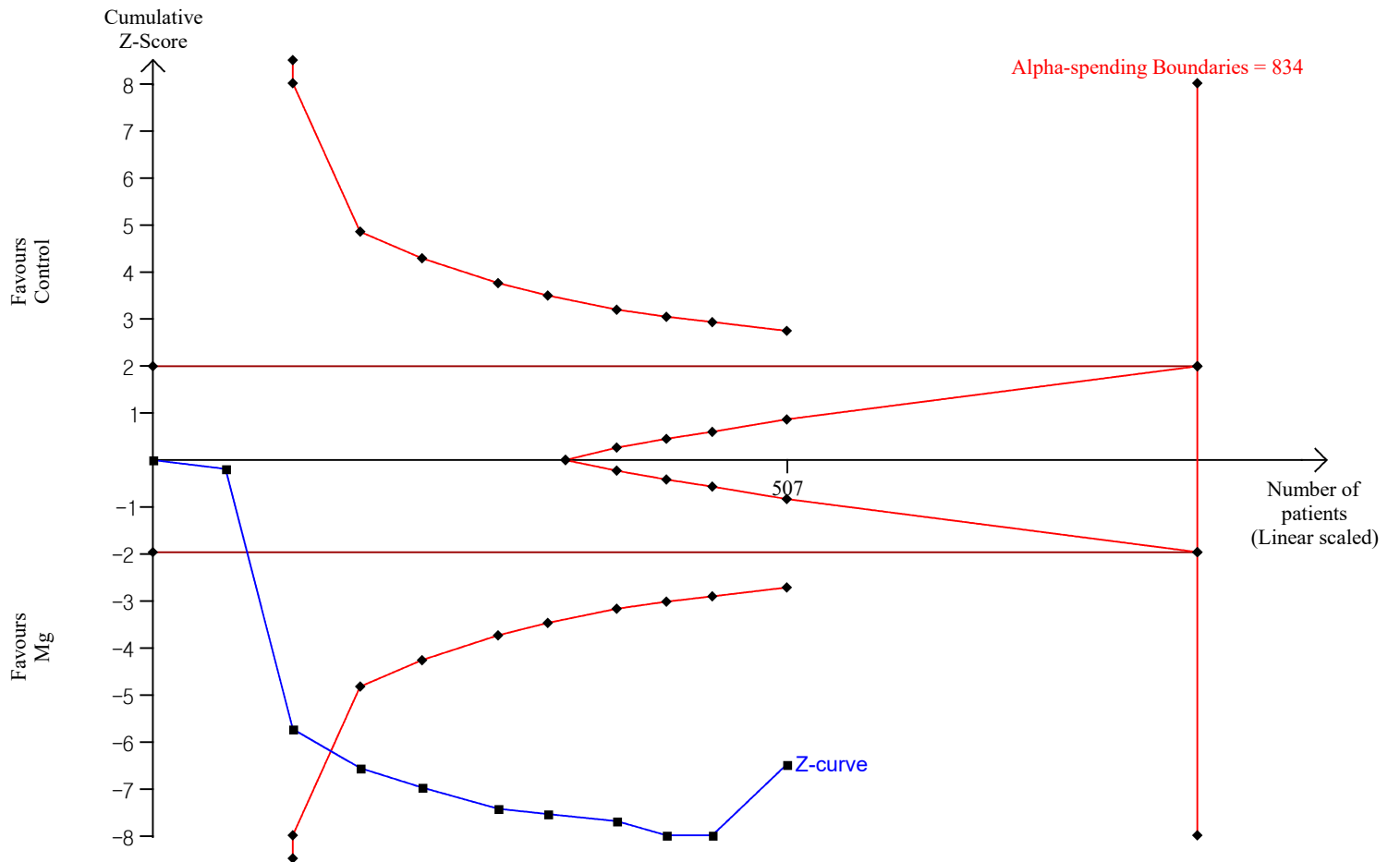

Supplement: Supplementary file 1 [file jpm-11-01273-s001.zip › S22.pdf]

Alpha-spending Boundaries is a Two-sided graph

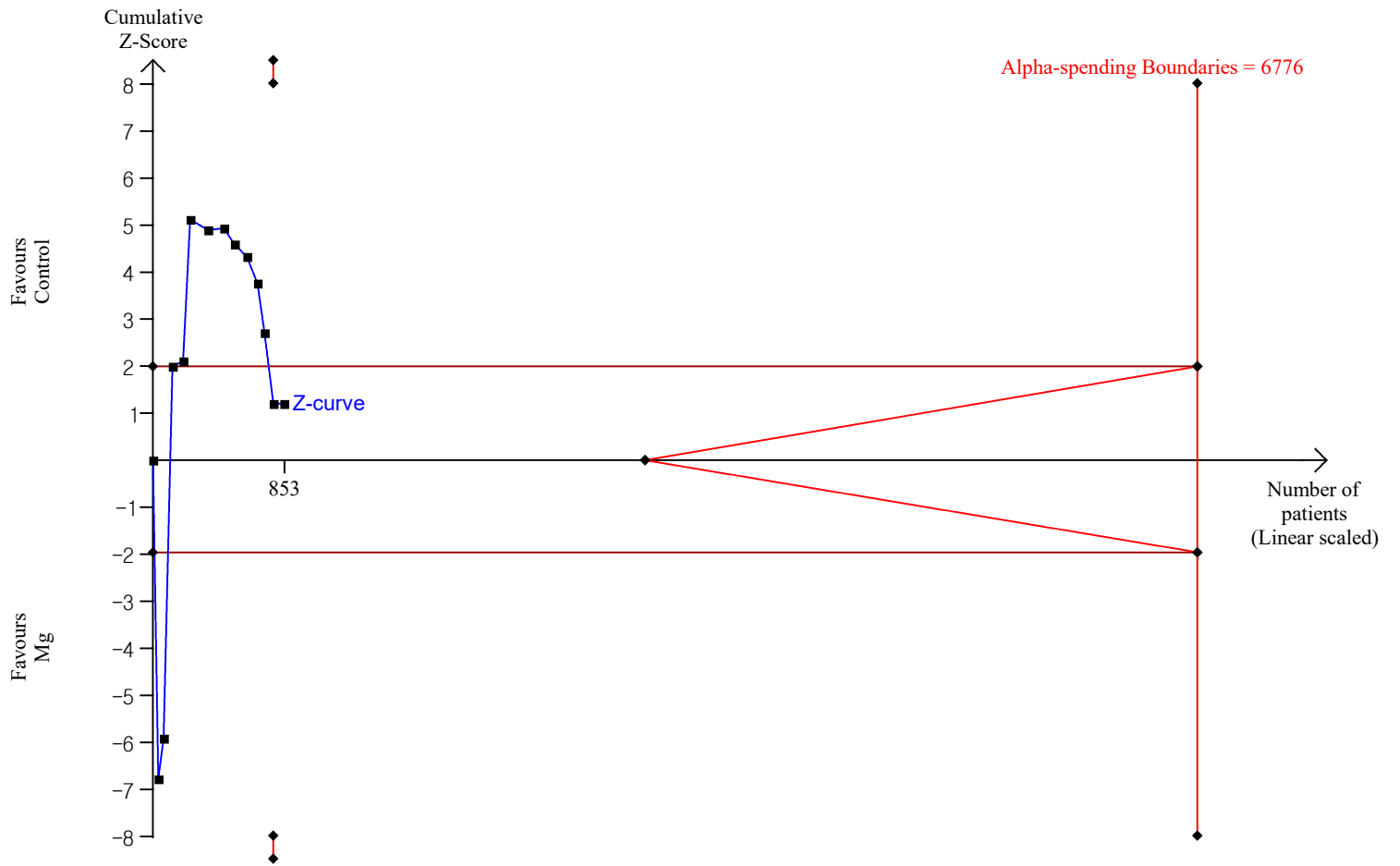

Supplement: Supplementary file 1 [file jpm-11-01273-s001.zip › S23.pdf]

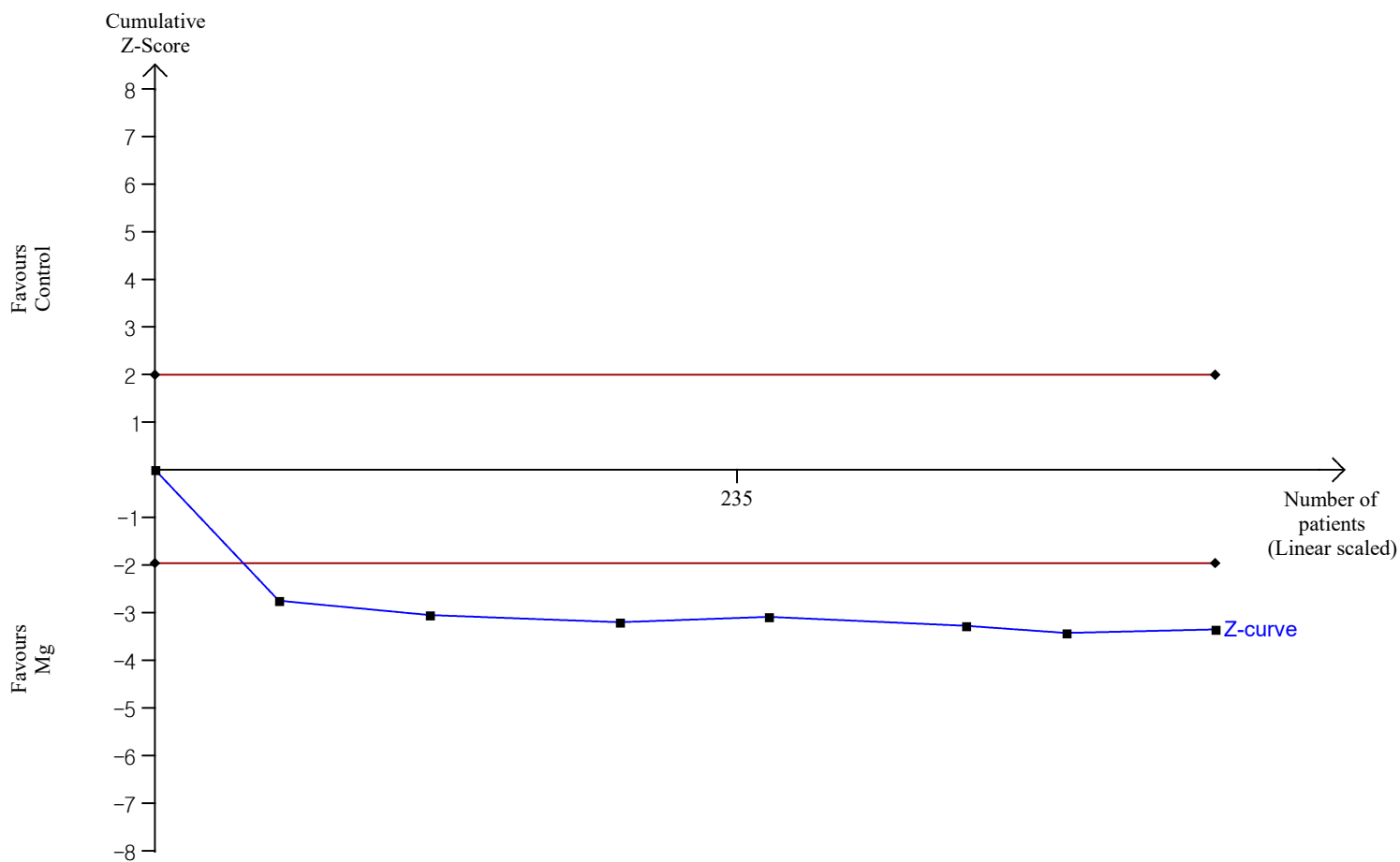

Supplement: Supplementary file 1 [file jpm-11-01273-s001.zip › S24.pdf]

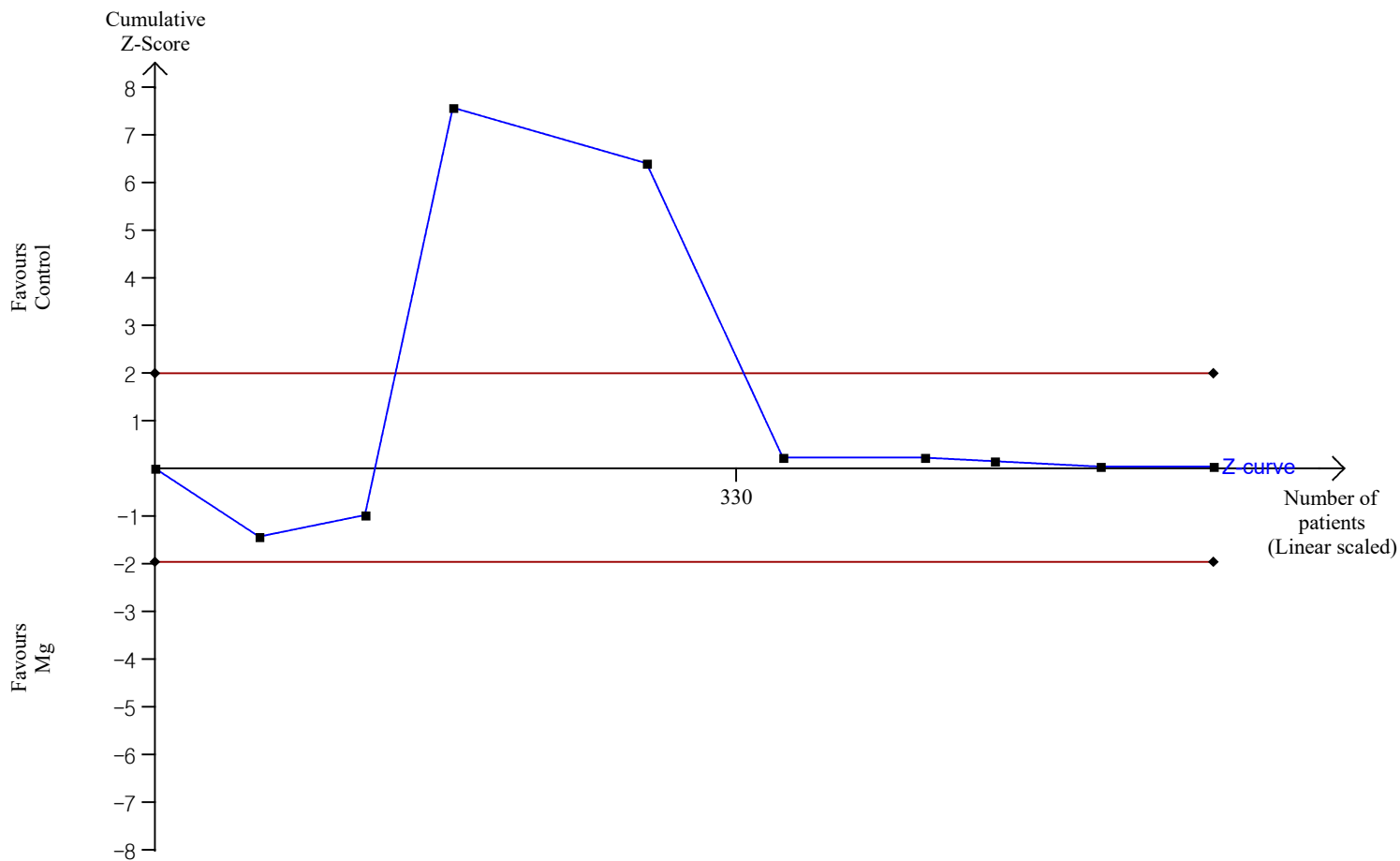

Supplement: Supplementary file 1 [file jpm-11-01273-s001.zip › S25.pdf]

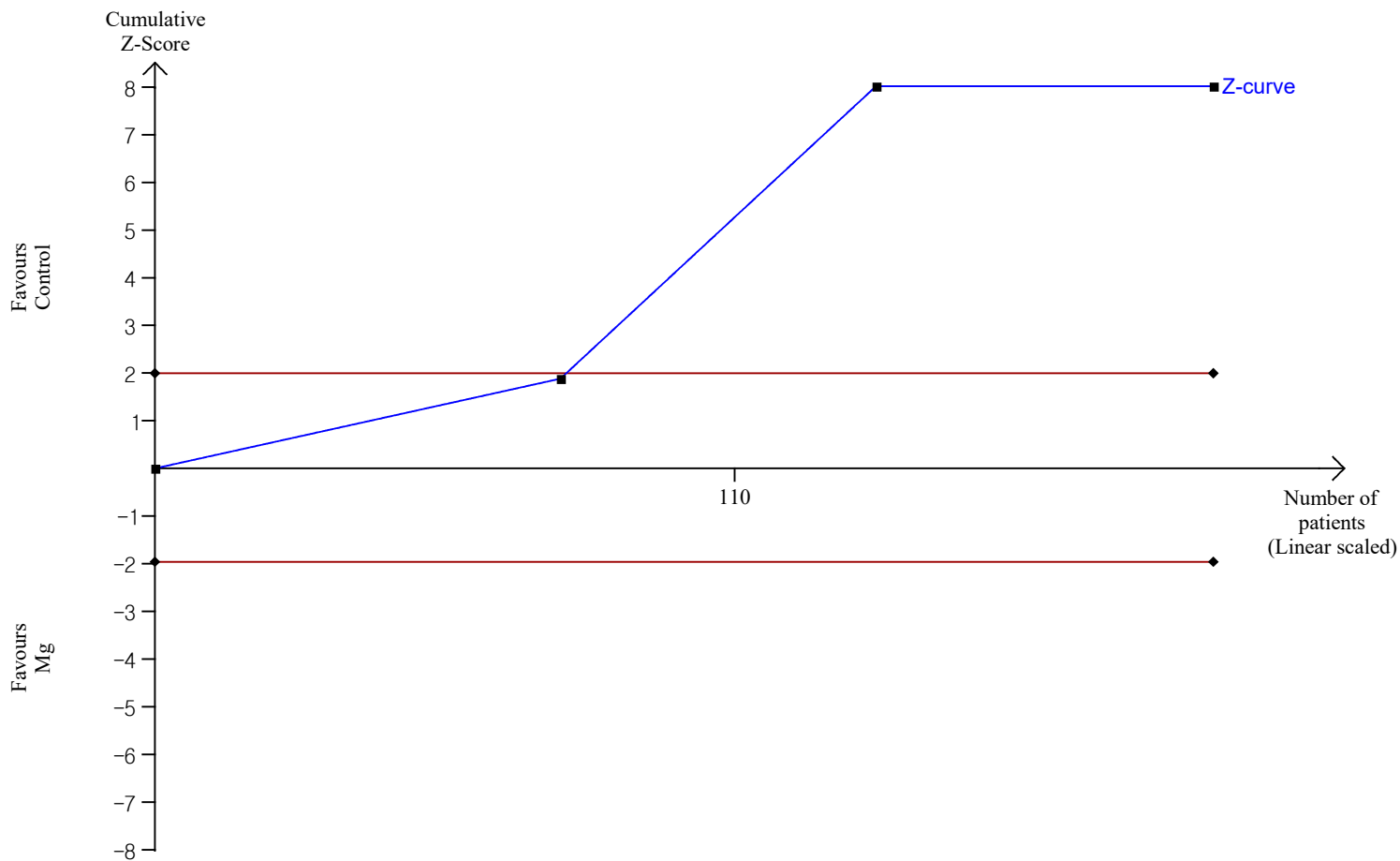

Supplement: Supplementary file 1 [file jpm-11-01273-s001.zip › S26.pdf]

# Alpha-spending Boundaries is a Two-sided graph

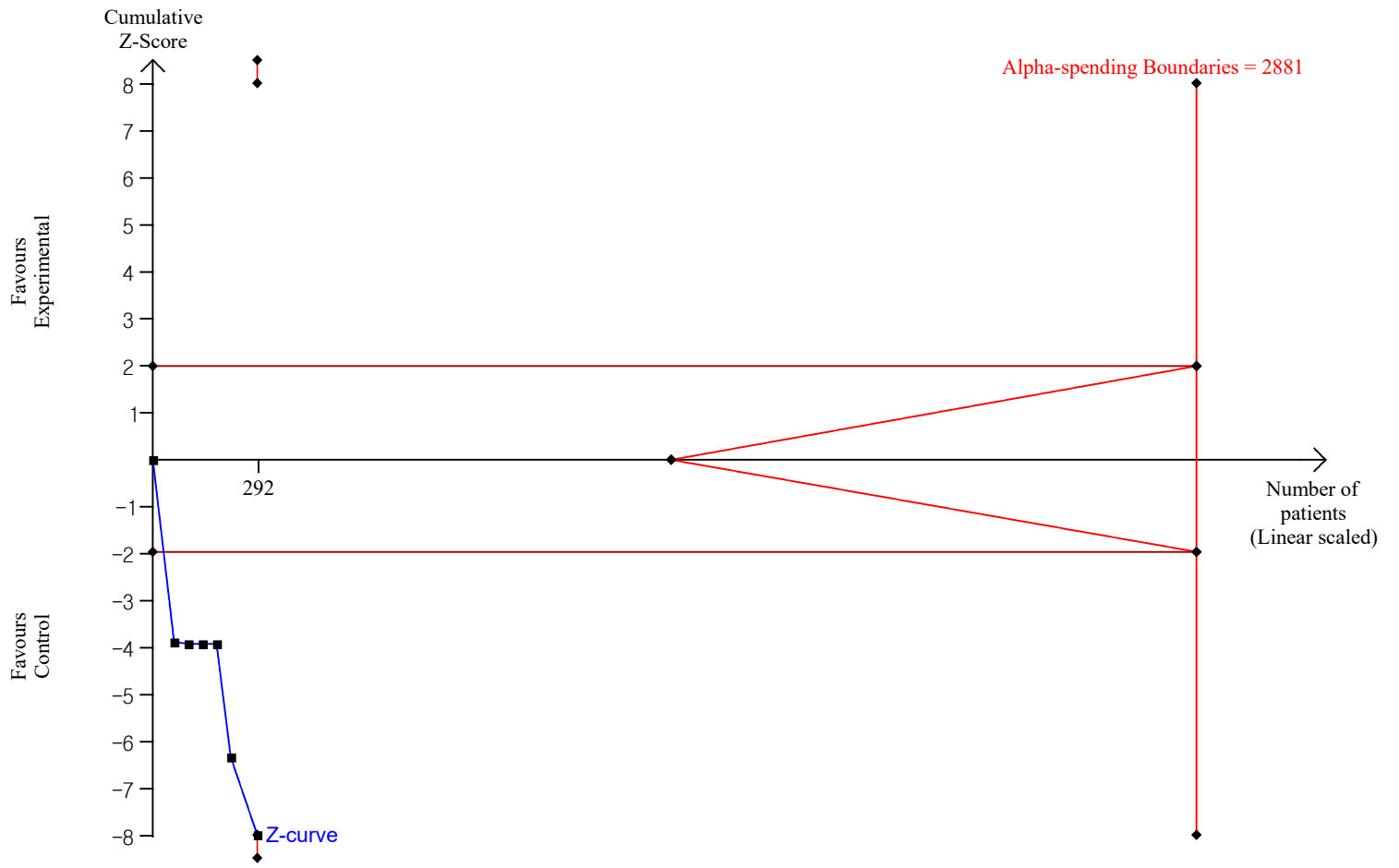

Supplement: Supplementary file 1 [file jpm-11-01273-s001.zip › S27.pdf]

# Alpha-spending Boundaries is a Two-sided graph

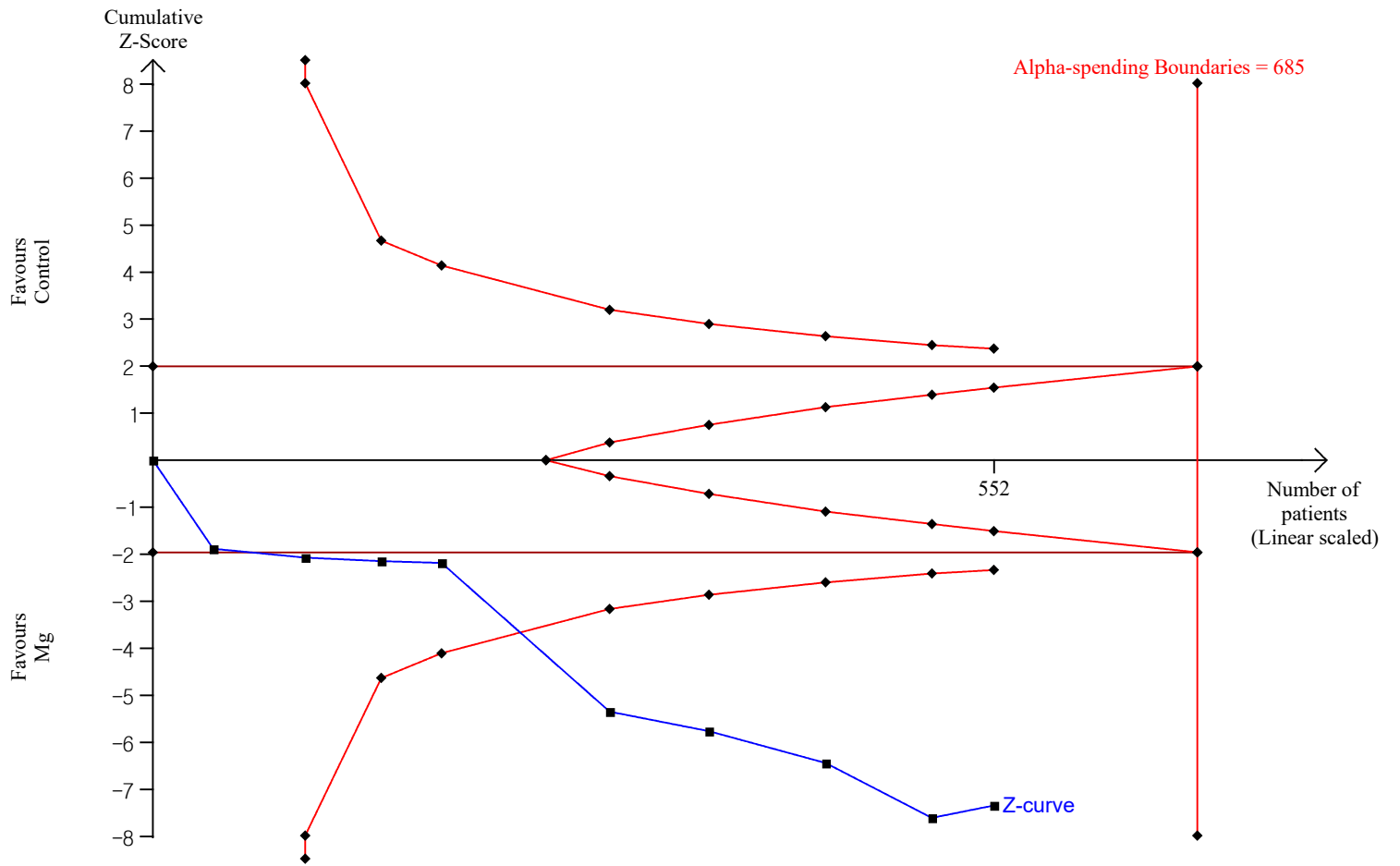

Supplement: Supplementary file 1 [file jpm-11-01273-s001.zip › S28.pdf]
